# A Multi-Strategy Improved Dung Beetle Optimizer for High-Dimensional Optimization and Engineering Applications

**DOI:** 10.3390/biomimetics11070485

**Published:** 2026-07-10

**Authors:** Shuxin Wang, Yinggao Yue, Mengji Xiong

**Affiliations:** 1 School of Intelligent Manufacturing, Shanghai Zhongqiao Vocational and Technical University, Shanghai 201514, China; 2 School of Electronic and Electrical Engineering, Wenzhou University of Technology, Wenzhou 325035, China

**Keywords:** high-dimensional optimization, dung beetle optimizer, whale optimization algorithm, seagull optimization algorithm, engineering application

## Abstract

When addressing high-dimensional complex optimization problems, the vanilla Dung Beetle Optimizer (DBO) suffers from slow convergence, frequent stagnation in local optima, and progressive degradation of population diversity. To overcome the above inherent defects, this paper proposes a multi-strategy hybrid improved DBO variant named the SWDBO, which incorporates three targeted enhancement modules. First, an adaptive population proportion strategy is developed to dynamically adjust the population sizes of rolling beetles, brood beetles, small beetles and thief beetles throughout iterations. More individuals are allocated for extensive global exploration at the early evolutionary stage, while more search agents are reserved for delicate local exploitation in later iterations, which maintains stable population diversity over the entire optimization process. Second, the bubble-net encircling and spiral predation mechanisms of the Whale Optimization Algorithm (WOA) are embedded into the position update formula of rolling beetles. This integration strengthens fine local search performance and accelerates the overall convergence rate. Third, a modified seagull optimization operator combined with Lévy random perturbation is introduced into the position updating rule of thief beetles. This improved jump mechanism optimizes individual movement trajectories and enables the algorithm to effectively escape local optimal traps. Numerical experiments are implemented on the 100-dimensional benchmark functions of CEC2017 and CEC2020. Moreover, the proposed SWDBO is validated on three classical constrained engineering optimization tasks, including three-bar truss design, ten-bar truss design and cantilever beam sizing optimization. Wilcoxon rank-sum tests statistically verify significant performance disparities between the SWDBO and competing optimizers. For the three structural engineering cases, the design solutions obtained by the SWDBO produce lighter structural mass while satisfying all constraint requirements. Overall experimental evidence proves that the proposed multi-strategy improvement framework can efficiently tackle high-dimensional numerical optimization and constrained engineering design problems, and the SWDBO exhibits prominent performance in balancing global exploration and local exploitation.

## 1. Introduction

With the rapid advancement of artificial intelligence, optimization techniques have attracted growing attention across engineering, management, economics and fundamental research. Driven by the iterative upgrade of the Internet of Things, intelligent manufacturing, aerospace engineering and civil structural design, practical optimization tasks are increasingly characterized by high complexity, high dimensionality and stringent constraint conditions [1,2]. In the big data context, high-dimensional optimization problems exhibit prominent features including intense nonlinearity, exponentially expanded search spaces and dramatically elevated computational complexity as dimensionality grows [3], which renders them a critical research frontier and longstanding challenge within the optimization community [4]. Conventional gradient-based optimization solvers fail to handle such complicated tasks efficiently. The deep fusion of artificial intelligence and industrial digitization further fuels the evolution of optimization theories [5,6]. Representative real-world high-dimensional optimization scenarios cover aerospace structural design, mechanical lightweight design, resource allocation scheduling, machine learning feature screening and chemical process parameter calibration [7]. Such practical tasks commonly suffer from high-dimensional variables, highly nonlinear objective functions, intricate feasible domains and a massive proliferation of local optima, resulting in severe curse-of-dimensionality effects [8,9]. Classical gradient-dependent deterministic optimizers heavily rely on the differentiability of objective functions, which frequently incur gradient vanishing and solution collapse in high-dimensional spaces, and thus cannot satisfy the demands of sophisticated engineering optimization [10,11].

As a class of gradient-free solvers with strong robustness and broad applicability, swarm intelligence optimization algorithms have emerged as mainstream tools for addressing high-dimensional nonlinear optimization problems [12]. Belonging to a vital subfield of artificial intelligence, swarm intelligence algorithms feature straightforward implementation workflows, fast convergence and a limited set of tunable hyperparameters. By imitating collective biological behaviors or natural physical laws, these algorithms conduct global optimum searching [13,14,15,16]. Popular metaheuristics proposed over recent decades include Particle Swarm Optimization (PSO), Grey Wolf Optimizer (GWO), Whale Optimization Algorithm (WOA), Sparrow Search Algorithm (SSA) and Dung Beetle Optimizer (DBO), all of which have been extensively deployed in diverse optimization tasks [17,18,19]. However, abundant existing research verifies that vanilla swarm intelligence optimizers suffer from universal high-dimensional degradation. As the variable dimension rises, the inherent tradeoff between global exploration and local exploitation collapses, accompanied by rapid population diversity attenuation, deteriorated convergence precision and frequent stagnation in abundant local optima [20]. In civil and mechanical engineering domains, truss and cantilever lightweight design are typical inequality-constrained optimization problems [21,22], where design variables are strongly coupled, constraint boundaries are convoluted and the search space is non-convex. Traditional enumeration and gradient-based approaches incur exponentially soaring computational overhead with increasing variable counts [23]. Accordingly, high-performance swarm intelligence optimizers are essential for low-cost structural lightweight design. In this context, reconstructing the architectures and search mechanisms of existing bionic algorithms to develop dimension-adaptive optimizers carries profound theoretical significance and substantial practical engineering value for both high-dimensional numerical optimization and constrained industrial design problems [24,25]. According to the No Free Lunch theorem, there is no universal algorithm that can solve all optimization problems. Thus, improving existing algorithms or developing new algorithms for specific problems has always been a research focus in the optimization field [26]. High-dimensional optimization problems have solution spaces expanding exponentially with dimensions, which put forward higher requirements for the global exploration and local exploitation abilities of algorithms [27]. Compared with various metaheuristic algorithms proposed in recent decades, the Dung Beetle Optimizer (DBO) is selected as the basic improvement framework of this paper with sufficient underlying logic and research necessity. First, different from traditional algorithms such as PSO, GWO and WOA that rely on a single population to complete both global and local searches, the DBO naturally constructs a hierarchical sub-population architecture based on four differentiated biological behaviors of dung beetles. It divides rolling, breeding, small and thief beetles into independent search units and separately assigns global exploration and local exploitation tasks, which endows it with an innate advantage to regulate search behaviors in stages at the underlying framework, matching the research demands of balancing search in high-dimensional spaces. Second, numerous existing studies have verified that the DBO possesses faster basic convergence speed and higher benchmark optimization accuracy than contemporary classic swarm intelligence algorithms. Nevertheless, most existing improved DBO variants are designed for low-dimensional path planning and simple numerical cases, and few systematic modifications are developed for harsh high-dimensional scenarios with 100 dimensions or above. There is a clear research gap in integrated multi-strategy optimization schemes suitable for ultra-high-dimensional and strongly constrained engineering problems. Third, compared with newly proposed biomimetic algorithms without hierarchical division such as BKA and BWO, the DBO has mature, decomposable iterative formulas for four types of individuals. There is no need to build a layered search framework from scratch; targeted optimization on different beetle modules can boost performance, leading to lightweight modification and clearer innovation orientation. In addition, engineering optimization tasks such as civil and mechanical lightweight design generally feature multiple constraints and non-convex feasible regions. The role-based search mechanism of the DBO is inherently suitable for searching constraint boundaries, which facilitates practical structural optimization including trusses and cantilever beams. Therefore, this paper takes the DBO as the core baseline and targets its three inherent drawbacks of rapid population diversity loss, weak local search and premature convergence during high-dimensional iterations.

The Dung Beetle Optimizer (DBO) is a novel swarm intelligence algorithm proposed by Xue et al. in 2023 [28]. It is inspired by four typical biological behaviors of dung beetles, including ball rolling, breeding, foraging of larvae and ball stealing [29]. Compared with PSO and GWO, the DBO has faster convergence and higher solution accuracy. However, similar to other swarm intelligence algorithms, the DBO also has drawbacks such as slow convergence, local optimum trapping and decreased population diversity in the later iteration stage [30]. Nevertheless, few studies have reported the application of the DBO and its improved variants to high-dimensional optimization problems [31]. Based on the above analysis, this paper proposes an improved Dung Beetle Optimizer (SWDBO) combining operators of the Seagull Optimization Algorithm and Whale Optimization Algorithm. Three main improvement strategies are adopted. First, an adaptive population ratio strategy is designed to dynamically adjust the quantity proportion of different types of dung beetles and enhance population diversity during iterations. Second, the bubble-net predation strategy of the WOA is integrated into the local search of rolling beetles to strengthen local search ability and accelerate convergence. Third, the spiral attack strategy of the improved Seagull Optimization Algorithm is introduced into the position update of breeding and thief beetles to reduce the probability of local optimum trapping. To evaluate the performance of the SWDBO, high-dimensional test functions from CEC2017 and CEC2020 are adopted for verification. The proposed algorithm is further applied to three engineering optimization problems, including three-bar truss design, ten-bar truss design and cantilever beam design. The results fully verify the feasibility and superiority of the SWDBO. Based on the above analysis, this article proposes an improved beetle optimization algorithm (SWDBO) based on the Seagull Optimization Algorithm operator and Whale Optimization Algorithm operator. The main improvement strategies of this algorithm include:

(1) Designing an adaptive population proportion strategy to dynamically adjust the proportion of different role beetles in the algorithm, in order to enhance the diversity of the population during the iteration process.
(2) Incorporating the Whale Optimization Algorithm’s bubble-net predation strategy into the local search phase of the rolling ball beetle enhances the algorithm’s local search capability and accelerates convergence speed.
(3) Introducing an improved Seagull Optimization Algorithm spiral attack strategy in the position updates of breeding and stealing beetles to reduce the probability of the algorithm falling into local optima.

## 2. Dung Beetle Optimizer

The DBO constructs population iteration rules based on four typical behaviors of dung beetles, namely, rolling dung balls, laying eggs and breeding, foraging of larvae and stealing dung balls. The whole population is divided into four groups: rolling beetles, brood beetles, small beetles and thief beetles [32]. All individuals cooperate to search for the global optimal solution. Let D be the dimension of the optimization problem and N be the total population size. The position vector of the i-th dung beetle at the t-th iteration is denoted as $X_{i}(t)=[X_{i1}(t),X_{i2}(t),\ldots ,X_{iD}(t)]$. $T_{\max}$ represents the maximum number of iterations. Ub and Lb are the upper and lower bounds of optimization variables [33]. The mathematical iteration formulas for four types of individuals in the original DBO are presented as follows.

### 2.1. Position Update of Rolling Beetles

Rolling beetles move along natural light to roll dung balls. Obstacles may block their moving paths [34]. The occurrence probability of obstacles is controlled by a random parameter η∈(0,1) and a threshold λ. The parameter α determines the moving direction, which is defined as

(1) $$\alpha =\left\{\begin{array}{c} 1,\;\;\;\eta >\lambda \\ -1,\;\;\;\eta <\lambda \end{array}\right.$$

When no obstacle exists, the beetle rolls the dung ball along a straight line. The position is updated as [35]

(2) $$\begin{array}{c} X_{i}(t+1)=X_{i}(t)+\alpha \cdot k\cdot X_{i}(t-1)+b\cdot \Delta X \end{array}$$

where k∈(0,0.02] is the deviation coefficient, b∈(0,1) is a constant, $\Delta X=|X_{i}(t)-X_{\mathrm{worst}}|$ represents light intensity, and $X_{\mathrm{worst}}$ is the worst position of the current population.

When encountering obstacles, the beetle dances in place to adjust the moving direction. θ∈[0,π] is the deflection angle during dancing. The position update formula is

(3) $$\begin{array}{c} X_{i}(t+1)=X_{i}(t)+tan\;\theta \cdot |X_{i}(t)-X_{i}(t-1)| \end{array}$$

When θ=π/2, tan θ is undefined, and the individual position remains unchanged.

### 2.2. Position Update of Brood Beetles and Brood Balls

Female brood beetles search for safe areas to bury dung balls and lay eggs [36]. The upper and lower bounds of the safe area are determined by the current local optimal position $X^{*}$:

(4) $$\left\{\begin{array}{c} Lb^{*}=\max\left(X^{*}(1-R),Lb\right)\\ Ub^{*}=\min\left(X^{*}(1+R),Ub\right) \end{array}\right.$$

where $X^{*}$ is the local optimal position in the current iteration, $R=1-t/T_{\max}$. $Lb^{*}$ and $Ub^{*}$ are the lower and upper bounds of the safe area.

R increases linearly with iterations and controls the shrinkage of the safe area. Female beetles lay eggs within the safe area [37]. The position update formula of brood balls is shown in Equation (7). If the brood ball position exceeds the safe area, the position is updated as

(5) $$\begin{array}{c} B_{i}(t+1)=X^{*}+b_{1}\cdot (B_{i}(t)-Lb^{*})+b_{2}\cdot (B_{i}(t)-Ub^{*}) \end{array}$$

where $b_{1}$ and $b_{2}$ are 1×D-dimensional independent random vectors. If the brood ball position crosses the boundary, boundary truncation is executed:

(6) $$B_{i}=\left\{\begin{array}{c} Lb^{*},\;{\;B}_{i}<Lb\\ Ub^{*},\;{\;B}_{i}>Lb \end{array}\right.$$

### 2.3. Position Update of Small Beetles

Brood balls hatch into small beetles. The foraging range of small beetles is defined near the global optimal position $X^{\mathrm{best}}$:

(7) $$\left\{\begin{array}{c} Lb^{best}=\max\left(X^{best}\cdot (1-R),Lb\right)\\ Ub^{best}=\min\left(X^{best}\cdot (1+R),Ub\right) \end{array}\right.$$

Small beetles search for food within the foraging range. The iteration formula is

(8) $$\begin{array}{c} X_{i}(t+1)=X_{i}(t)+C_{1}\cdot (X_{i}(t)-Lb^{best})+C_{2}\cdot (X_{i}(t)-Ub^{best}) \end{array}$$

where $C_{1}$ is a random vector following normal distribution, and $C_{2}\in (0,1)$ is a random vector [38].

### 2.4. Position Update of Thief Beetles

Thief beetles steal dung balls near the global optimal position. The iteration formula is

(9) $$\begin{array}{c} X_{i}(t+1)=X^{best}+S\cdot g\cdot (|X_{i}(t)-X^{*}|+|X_{i}(t)-X^{best}|) \end{array}$$

where S is a constant, and g is a 1×D-dimensional normal random vector. The original DBO adopts a fixed proportion of four types of individuals throughout iterations. It cannot dynamically adjust population structure according to the search process [39]. This leads to imbalance between global exploration and local exploitation in high-dimensional optimization. In addition, rolling beetles have single movement modes, and thief beetles lack long-distance perturbation capability [40]. These are the main reasons for the poor performance of the original DBO in high-dimensional optimization.

## 3. Multi-Strategy Hybrid Improved Dung Beetle Optimizer (SWDBO)

Aiming at three main defects of the original DBO, including fixed population proportion, weak local search and local optimum trapping, three improvement strategies are adopted in this paper. An adaptive population ratio strategy, whale bubble-net local optimization strategy, and improved seagull operator combined with Lévy flight perturbation strategy are integrated to modify the algorithm from population structure, local exploitation and global escape perspectives. The improved algorithm is named the SWDBO. To address three mutually independent inherent drawbacks of the original Dung Beetle Optimizer, i.e., fixed population composition, inefficient local exploitation and premature convergence on high-dimensional multimodal landscapes, this paper devises three improvement strategies, including adaptive population ratio, WOA bubble-net local search and modified seagull operator with Lévy perturbation, which possess distinct hierarchical complementary logic. The adaptive population ratio strategy dynamically allocates individuals for global exploration and local exploitation at the macro population level, solving the fundamental imbalance of search resources throughout iterations and providing a balanced population foundation for the other two operators to function. The WOA bubble-net mechanism only acts on rolling beetles and enhances precise mining near optimal regions via shrinking encircling and spiral search modes, making up for the slow convergence in the middle search stage and taking full advantage of local search agents allocated by the adaptive strategy. The modified seagull operator integrated with long-range Lévy jumps is specially embedded into thief beetles, endowing the algorithm with cross-domain jumping capability to escape dense local optimum traps and compensating the deficiency that the first two strategies only support short-range searching without global escape functions. The three strategies target three independent optimization dimensions of population structure, local convergence and global escape without overlapping functions and separate defect-oriented targets. They form a complete collaborative loop of macro control, precise exploitation and global escape from top to bottom, which jointly eliminates all core performance deficiencies of the original DBO from the source.

### 3.1. Adaptive Population Ratio Strategy

The original DBO maintains a fixed number of four types of beetles during iterations. In the early stage, insufficient rolling beetles limit the global exploration range [41]. In the later stage, the quantity of brood beetles and thief beetles is too small to conduct refined search near optimal regions. Population diversity declines rapidly in high-dimensional optimization. Referring to the adaptive allocation idea of the improved Sparrow Search Algorithm, a nonlinear dynamic coefficient a(t) is constructed to dynamically adjust the number of rolling beetles $N_{1}$, brood beetles $N_{2}$, small beetles $N_{3}$ and thief beetles $N_{4}$. The total population satisfies $N=N_{1}+N_{2}+N_{3}+N_{4}$. The dynamic adjustment coefficient is defined as

(10) $$\begin{array}{c} a(t)=0.15\cdot \frac{-2t}{T_{max}}-0.1\cdot rand+0.1 \end{array}$$

where rand∈[0,1] is a uniform random number. The population is allocated adaptively as follows [42]:

(11) $$\left\{\begin{array}{c} N_{1}=a(t)\times N\\ N_{2}=0.2\times (1-a(t))\times N\\ N_{3}=0.25\times (1-a(t))\times N\\ N_{4}=N-N_{1}-N_{2}-N_{3} \end{array}\right.$$

In the early iteration stage, t is small and a(t) is large. More rolling beetles are arranged to strengthen global exploration and fully traverse the high-dimensional search space. In the later stage, t approaches $T_{\max}$ and a(t) decreases gradually. The proportion of brood beetles, small beetles and thief beetles increases. These individuals focus on refined local search near optimal regions [43]. This strategy breaks the limitation of fixed proportion, balances the number of individuals for exploration and exploitation in the whole iteration process, delays the loss of population diversity in high-dimensional environments, and optimizes the basic performance of the algorithm from the perspective of population structure.

### 3.2. Integration of Whale Optimization Algorithm Bubble-Net Predation Strategy

Original rolling beetles only move in straight lines or change directions. The single movement mode leads to slow convergence when approaching optimal solutions. The dual search mechanisms of the WOA, namely, shrinking encircling and spiral foraging, are used to reconstruct the position update formulas of rolling beetles to replace Equations (2) and (3) [44,45]. A random warning parameter $R_{2}\in [0,1]$ is used to select different search modes, and the safety threshold ST∈[0.5,1].

If $R_{2}<ST$, the shrinking encircling mechanism is executed:

(12) $$\begin{array}{c} X_{i}(t+1)=X^{best}(t)-A\cdot |C\cdot X^{best}(t)-X_{i}(t)| \end{array}$$

If $R_{2}\geq ST$, the spiral surrounding foraging mechanism is activated:

(13) $$\begin{array}{c} X_{i}(t+1)=X^{best}(t)+D\cdot e^{l}\cdot cos(2\pi l) \end{array}$$

The relevant parameters are defined as

(14) $$\left\{\begin{array}{c} A=2\times \left(1-{\left(\frac{t}{T_{max}}\right)}^{2}\right)\times r_{1}-r_{1}\\ C=2\times r_{2}\\ D=\left|X^{best}(t)-X_{i}(t)\right|\\ l\in [-1,1] \end{array}\right.$$

where $r_{1},r_{2}\in [0,1]$ are uniform random numbers. The convergence factor A adopts a square decreasing mode [46]. In early iterations, A has a large value to expand the local search range. In later iterations, A decreases rapidly to realize refined local exploitation. After integrating the dual predation mechanisms of the WOA into rolling beetle update rules, the neighborhood search paths of individuals are enriched [47]. The local exploitation accuracy is greatly improved, and the convergence speed for high-dimensional functions is accelerated.

### 3.3. Integration of Improved Seagull Optimization Operator and Lévy Flight Perturbation

Original thief beetles only conduct a short-distance search around the global optimal position. They lack the perturbation capability to escape local optima and easily suffer from premature convergence in high-dimensional multimodal optimization [48]. The spiral attack operator of the improved Seagull Optimization Algorithm and the Lévy random step are combined to construct a new position update formula for thief beetles, which replaces Equation (9) [49]. The basic spiral coordinate transformation of seagulls is

(15) $$\left\{\begin{array}{c} x=r\cos\theta \\ y=r\sin\theta \\ z=r\theta \\ r=\mu e^{\theta \cdot v},\mu =1,v=0.1 \end{array}\right.$$

where θ∈[0,2π]. An adaptive variation coefficient $A_{\mathrm{soa}}=f_{e}-\frac{t\cdot f_{c}}{T_{\max}}$ is constructed, and $f_{c}$ is a control constant. The basic position components of seagulls are

(16) $$\left\{\begin{array}{c} G_{s}(t)=A_{soa}\cdot X_{i}(t)\\ M_{s}(t)=B\cdot \left(X^{best}-Xi(t)\right),B=2Asoa^{2}\cdot r_{d},r_{d}\in [0,1] \end{array}\right.$$

where $r_{d}$ is a random coefficient. An adaptive weight ω based on sine function is introduced:

(17) $$\begin{array}{c} \omega =\omega_{min}+(\omega_{max}-\omega_{min})\cdot sin(\pi +\frac{t\pi }{2T_{max}}) \end{array}$$

where $\omega_{\max}$ and $\omega_{\min}$ are the upper and lower bounds of the weight. The Lévy flight random step L(λ) follows the power law distribution

(18) $$\begin{array}{c} L(s,\lambda )=\frac{\sigma_{\mu }\cdot \mu }{|\nu {|}^{1/\beta }},1<\lambda \leq 3 \end{array}$$

(19) $$\begin{array}{c} \sigma_{\mu }={\left(\frac{\Gamma (1+\beta )sin\;(\pi \beta /2)}{\Gamma [(1+\beta )/2]\cdot \beta \cdot 2^{(\beta -1)/2}}\right)}^{\frac{1}{\beta }} \end{array}$$

where $\mu ∼N(0,\sigma_{\mu }^{2})$, ν∼N(0,1), and Γ denotes the Gamma function. Combining the above operators, the improved position update formula for thief beetles is obtained:

(20) $$\begin{array}{c} X_{i}(t)=(G_{s}(t)+M_{s}(t))\cdot xyz+\omega \cdot L(\lambda )\cdot X^{best} \end{array}$$

The improved thief beetles combine the spiral surrounding search of seagulls and long-distance jump of Lévy flight. They can perform a refined search near optimal regions and jump out of local optimal areas via large Lévy steps. The global escape capability is significantly enhanced, and the premature convergence problem in high-dimensional multimodal optimization is relieved. The position update formulas of brood beetles and small beetles remain consistent with the original DBO.

### 3.4. Implementation Steps and Pseudo-Code of SWDBO

The algorithm implementation steps of the SWDBO are as follows:

The SWDBO retains the population classification framework of the original DBO. Only the position update rules of rolling beetles and thief beetles are modified. The update formulas of brood beetles and small beetles are unchanged. The detailed iteration steps are as follows.

Step 1: Initialize parameters and population. Input optimization dimension, population size, maximum iteration number, variable bounds and hyperparameters of improvement strategies. Randomly initialize the population within feasible regions. Calculate the fitness of all individuals, and select the global optimal position $X^{\mathrm{best}}$, local optimal position $X^{*}$ and the worst individual position. Set the iteration counter t=1.

Step 2: Divide the four types of beetles adaptively. Calculate the dynamic coefficient and the quantity of each type of beetle via Equations (10) and (11) at the start of each iteration. Realize dynamic resource allocation for global exploration in early iterations and local exploitation in later iterations.

Step 3: Update rolling beetles using the whale bubble-net strategy. Select the shrinking encircling or spiral update formula according to $R_{2}$, and execute boundary truncation.

Step 4 and Step 5: Update brood beetles and small beetles following Equations (4)–(8) of the original DBO, and process boundary constraints.

Step 6: Update thief beetles using the improved seagull operator combined with Lévy flight in Equations (15)–(20), and process boundary constraints.

Step 7: Select individuals greedily and update optimal solutions. Compare the fitness of new and old positions after all individuals are updated, and retain better positions. Refresh the global and local optimal solutions.

Step 8: Judge iteration termination. Set t=t+1. If the maximum iteration number is not reached, enter the next iteration; otherwise, output the optimal solution.

The pseudocode of the SWDBO algorithm is shown in Algorithm 1.
**Algorithm 1.** Implementation of the proposed SWDBO algorithm**Input**: Maximum iteration $T_{max}$, population size N, optimization dimension D, variable lower bound Lb, variable upper bound Ub, relevant hyperparameters of improved strategies **Output**: Global optimal position $X^{best}$, global optimal fitness $F_{best}$Initialize the initial population X within the range [Lb,Ub]$\mathrm{Calculate}\;\mathrm{the}\;\mathrm{fitness}\;\mathrm{of}\;\mathrm{all}\;\mathrm{individuals},\;\mathrm{select}\;X^{best}$, $X^{*}$, $X_{worst}$, set iteration counter t=1$\mathrm{while}\;t<T_{max}$ doCalculate dynamic coefficient a(t) by Equation (10), divide the population into $N_{1}$, $N_{2}$, $N_{3}$, $N_{4}$ via Equation (11)$\mathrm{Generate}\;\mathrm{random}\;\mathrm{factor}\;R_{2}$, set safety threshold ST=0.7**// Update** rolling **beetles (***N*_1_ **individuals)**
**for each** rolling beetle **do**$\mathrm{if}\;R_{2}<ST\;$**then Update** position using Equation (12)
**else** Update position using Equation (13)**end if**Enforce **boundary** constraints on individual positionend **for****// Update brood** beetles **(***N*_2_ **individuals)**
**for** each brood beetle **do**Update position using Equations (4)–(6), enforce boundary constraintsend for**//** Update **small beetles (***N*_3_ **individuals)**
**for** each small beetle **do**Update position using Equations (7) and (8), enforce boundary constraints**end for****//** Update **thief beetles (***N*_4_ **individuals)**
**for** each thief beetle **do**Update position using Equations (15)–(20), enforce boundary constraints**end** forCalculate new fitness values for all updated individualsUpdate population by greedy selection strategy$\mathrm{Refresh}\;X^{best}$ and $X^{*}$ according to current populationt=t+1end **while**$\mathrm{return}\;X^{best}$, $F_{best}$

### 3.5. Algorithm Complexity Analysis

Let N be the total population size, $T_{\max}$ be the maximum iteration number and D be the optimization dimension. The time complexity of the original DBO consists of population initialization O(N⋅D), fitness calculation O(N) and position update of four types of individuals O(N⋅D). The overall time complexity of the original DBO is $O(T_{\max}\cdot N\cdot D)$.

The complexity of improved parts in the SWDBO is analyzed as follows. First, the adaptive population ratio only involves simple arithmetic operations to calculate $N_{1}$ to $N_{4}$. No additional loops or position updates are added, so no extra complexity is introduced. Second, the whale bubble-net formulas for rolling beetles have the same computation magnitude as original update rules. The computational load remains unchanged. Third, the improved seagull operator and Lévy flight are single operations for D-dimensional vectors. The complexity of a single individual update is still O(D), and no nested loops are added.

In summary, the overall time complexity of the SWDBO is still $O(T_{\max}\cdot N\cdot D)$, which is consistent with the original DBO. The improvement strategies do not significantly increase computational overhead and CPU running time. The proposed algorithm balances optimization performance and computational efficiency.

## 4. Comparative Experiments and Results Analysis

All numerical simulations and engineering case experiments in this paper are conducted under a unified hardware and software environment. The simulation platform adopts MATLAB R2023b with an Intel Core i7 processor and 16 GB RAM. The proposed SWDBO and all comparative algorithms share identical population size, maximum iterations, termination criteria and random seeds. Each test runs 30 independent replicates, and redundant background system processes are closed during the experiments to eliminate performance bias caused by resource competition. The average runtime and peak memory consumption of each algorithm are recorded synchronously throughout the tests. Combined with accuracy metrics including best fitness, mean value and standard deviation, a comprehensive performance evaluation covering both optimization precision and practical computational overhead is implemented. Two sets of high-dimensional benchmark functions from CEC2017 and CEC2020 are selected for comparison. The comparative algorithms include the original DBO, PSO [50], Black-winged Kite Algorithm (BKA) [51], GWO [52], Honey Badger Algorithm (HBA) [53], WOA [54] and Beluga Whale Optimization (BWO) [55]. The parameters of all the comparative algorithms are set according to the original studies. Each algorithm runs independently for 30 times to guarantee statistical significance. Unified parameter settings: population size N=30, maximum iteration number $T_{\max}=1000$, function dimension Dim=100. Three evaluation indices, including best value, mean value and standard deviation (Std), are recorded. The original DBO uses fixed population proportion. PSO adopts classic inertia weight and learning factors. The parameters of the GWO, WOA and other algorithms follow default settings in the original studies. The hyperparameters of the SWDBO are determined by the proposed formulas and remained unchanged during the experiments.

### 4.1. CEC2017 High-Dimensional Test Functions

**Figure 1:**
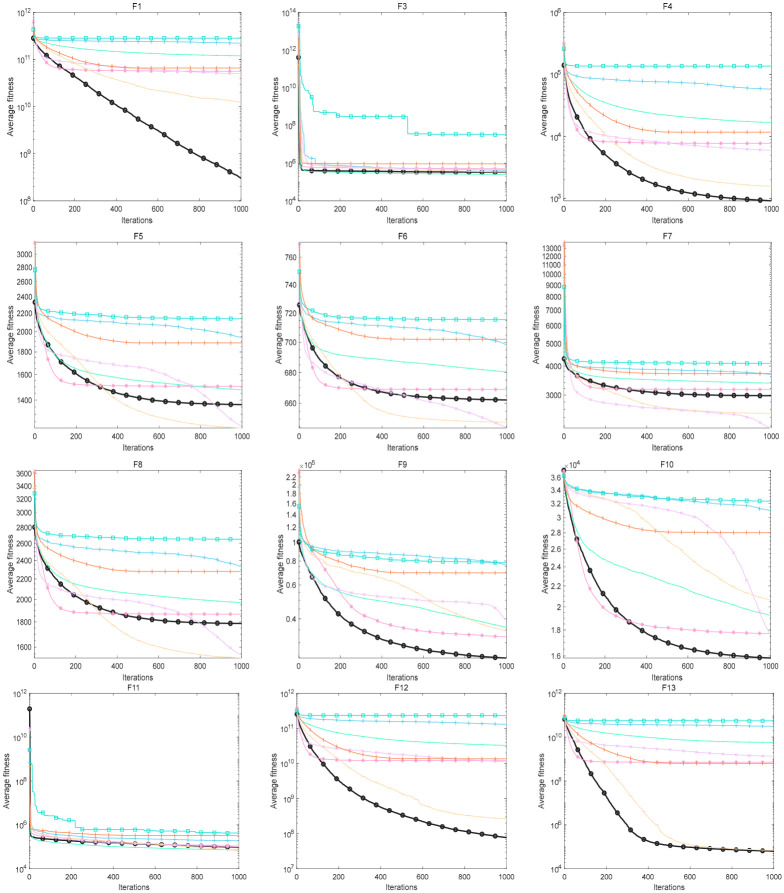

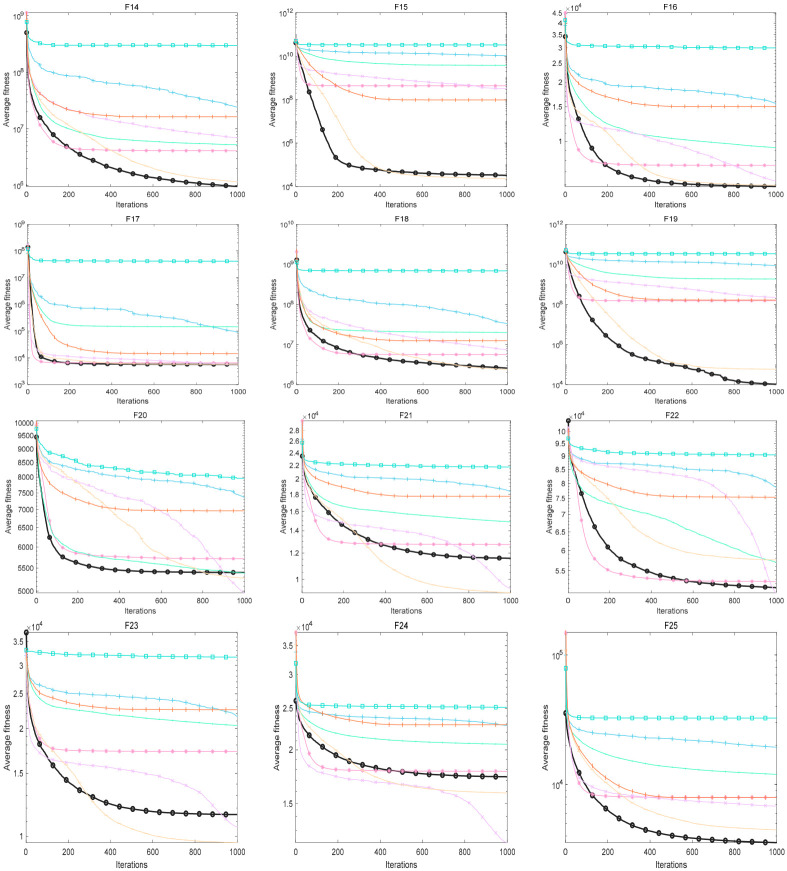

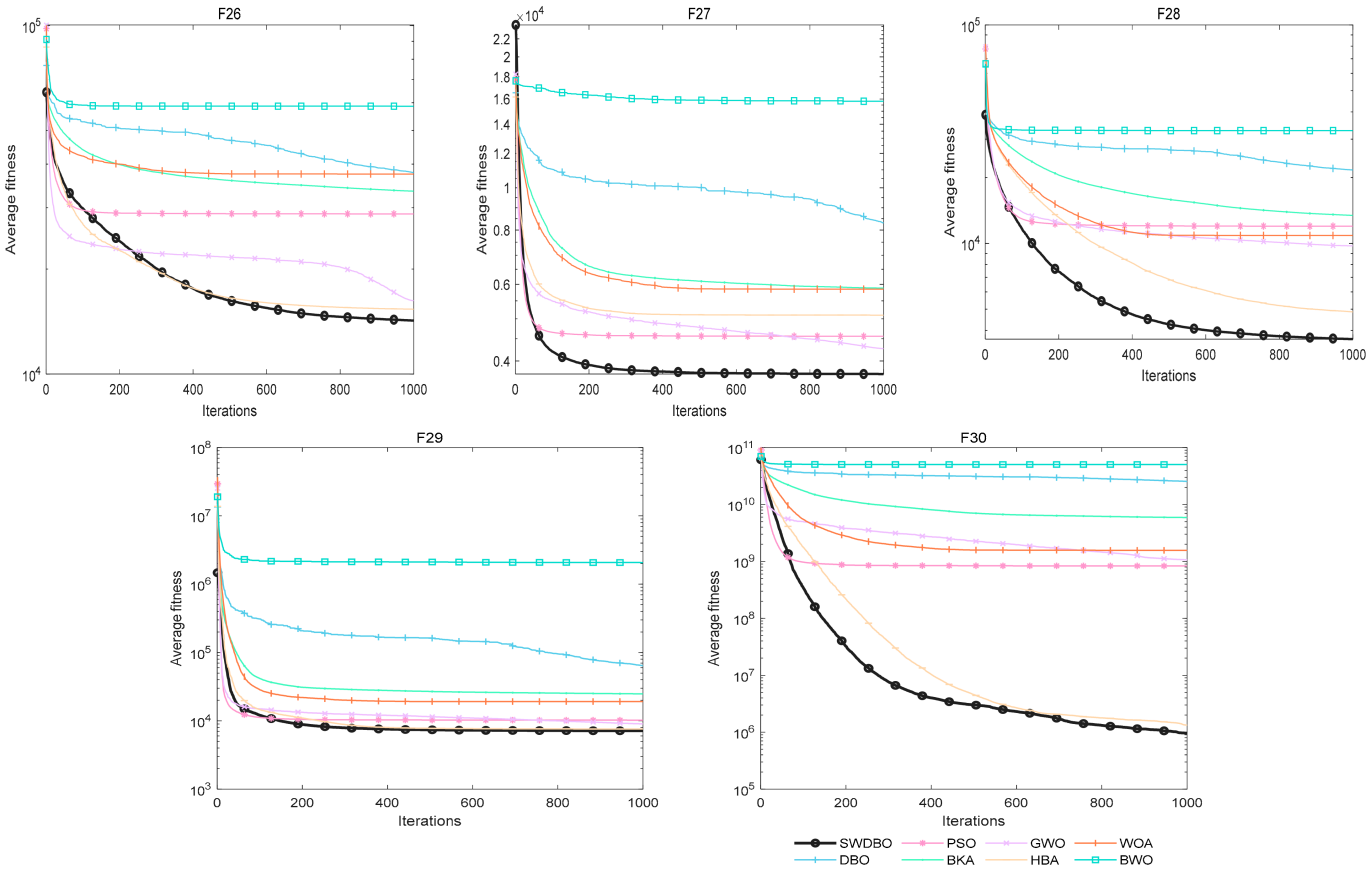
Comparison of average convergence curves of CEC2017 test set algorithms.

**Figure 2:**
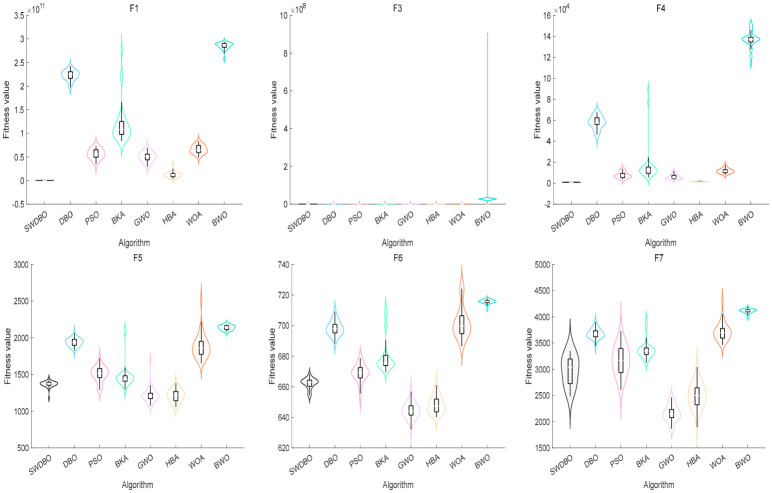

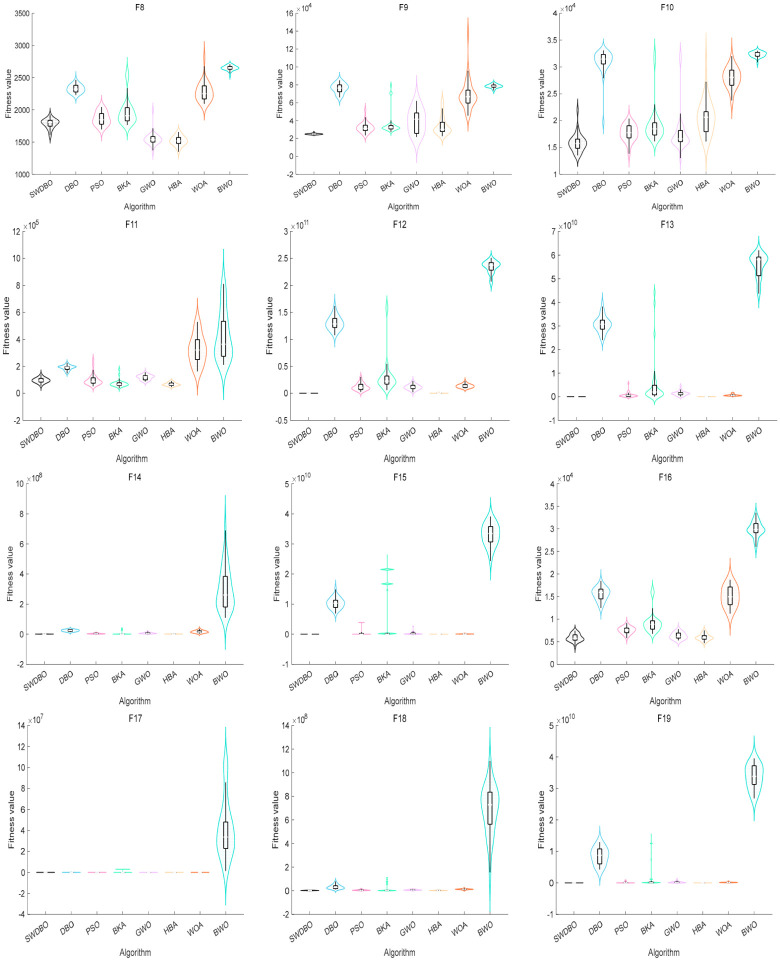

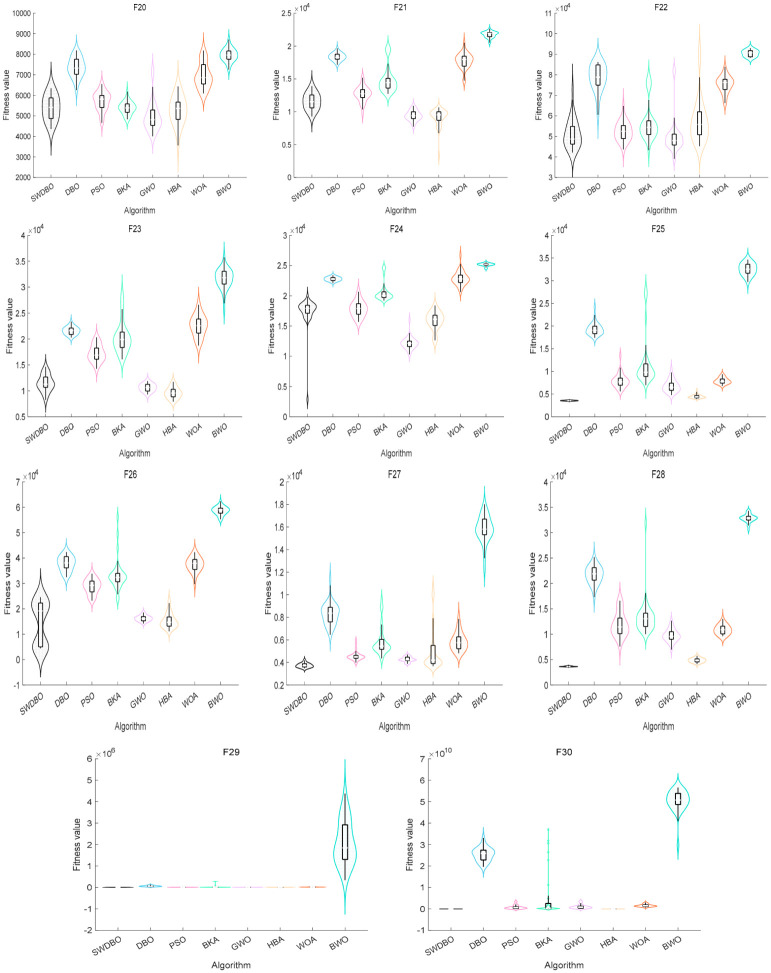
Comparison of violin plots of CEC2017 test set algorithm data.

**Figure 3:**
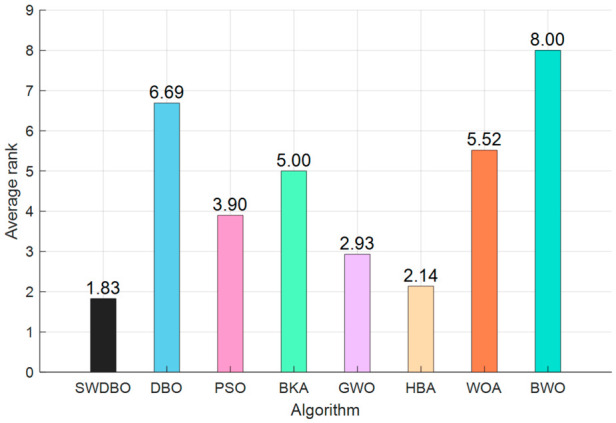
Comparison of average ranking of 8 algorithms in the CEC2017 test set.

CEC2017 contains 30 standard test functions, which are divided into three categories. F1 and F3 are unimodal functions with a single global optimum and no local optima. They are used to test the local exploitation ability and convergence speed of algorithms. F4–F10 are basic multimodal functions with numerous local optima, which mainly verify the capability to escape from local optima. F11–F30 are hybrid composite functions processed by coordinate translation, random rotation and nonlinear superposition. Their search spaces are distorted and irregular. The curse of dimensionality is severe under 100-dimensional settings, which can effectively distinguish the comprehensive performance of different algorithms. Table 1 lists the optimization results of the SWDBO and seven comparative algorithms on CEC2017 test functions. Table 2 presents the Wilcoxon rank-sum test results. Figure 1 shows the average convergence curves of all algorithms. Figure 2 displays the violin plots of the experimental data. Figure 3 illustrates the average ranking of eight algorithms.

According to the results of 30 independent runs on all 30 functions, the SWDBO outperforms the other seven comparative algorithms in terms of best value and mean value on most test functions. Only a few functions show slightly worse results than the BKA. For unimodal functions, the original DBO, PSO, GWO and other traditional swarm intelligence algorithms suffer from insufficient local exploitation individuals in later iterations under 100-dimensional spaces. Their obtained optimal values deviate greatly from theoretical optima. Benefiting from the dual search mechanisms of the WOA, the SWDBO optimizes the update rules of rolling beetles and strengthens the refined search near optimal regions. It achieves a remarkable improvement of convergence accuracy on unimodal problems. For multimodal and composite functions, thief beetles of the original DBO only conduct a short-distance search around optimal positions and lack long-distance jump capability. The algorithm is easily trapped into local optima. PSO and WOA also have insufficient global perturbation, leading to large result fluctuations and high standard deviations in multiple runs. Combining the improved seagull operator and Lévy flight perturbation, thief beetles of the SWDBO perform alternating short-range refined search and long-range random jump. They can effectively escape from dense local optima. Meanwhile, the adaptive population ratio strategy dynamically allocates the number of different beetles in the whole iteration process. More rolling beetles are arranged in early iterations to traverse the entire search space and avoid missing optimal regions. More brood beetles and thief beetles are allocated in later iterations to focus on local exploitation. Population diversity is maintained throughout iterations. Therefore, the SWDBO has much lower standard deviations than comparative algorithms and presents superior robustness.

From the perspective of convergence curves, the original DBO, PSO, GWO and other algorithms tend to be flat after 150–250 iterations. The fitness no longer decreases obviously, which indicates typical premature convergence. In contrast, the convergence curve of the SWDBO drops rapidly in the early stage. Most functions complete most of the search tasks within the first 50 iterations. The curve continues to decline slowly in later iterations without obvious stagnation. The three improvement strategies work cooperatively. The adaptive population ratio realizes dynamic allocation of search resources. The whale optimization mechanism accelerates local convergence. The hybrid perturbation of the seagull operator and Lévy flight helps the algorithm escape from local optima continuously. The SWDBO balances fast global search in early iterations and refined local exploitation in later iterations, and achieves simultaneous improvement of convergence speed and accuracy. Traditional algorithms such as PSO and WOA are seriously affected by the curse of dimensionality, while the SWDBO effectively relieves the adverse effects of high dimensions and maintains stable optimization performance.

Combining the convergence curves and temporal population diversity curves of all unimodal, multimodal and composite functions in CEC2017, we horizontally compare the dynamic exploration/exploitation balance of the DBO, PSO, GWO and other competitors and deeply reveal the internal mechanism in which three coordinated strategies of the SWDBO improve convergence behavior. Most comparative algorithms adopt fixed population frameworks or single weight adjustment rules. Insufficient global search agents in early iterations easily lead to missed high-quality optimal regions; severe population homogenization occurs in the middle and late stages, and the algorithms fail to conduct continuous precise search after falling into local optima, resulting in flat stagnation on convergence curves in advance. In contrast, the adaptive population ratio mechanism of the SWDBO nonlinearly adjusts the number of four beetles with iterations. A larger number of rolling beetles are allocated at the early stage to complete full global exploration. In the middle phase, the dual search modes of the WOA bubble-net are utilized to carry out multi-layer local mining around potential optima and rapidly reduce fitness values. In the late iteration, the seagull operator combined with long Lévy jumps continuously breaks local traps to mine superior solutions. The three-layer regulation logic cooperates from top to bottom and realizes smooth dynamic switching between exploration and exploitation throughout the optimization process, which serves as the core internal reason why SWDBO outperforms all competitors in convergence speed and solution accuracy on the entire CEC2017 benchmark suite.

The Wilcoxon rank-sum test results show that the *p*-values between the SWDBO and DBO, PSO, GWO, WOA, HBA, and BWO are all less than 0.05 except for individual functions compared with the BKA. It proves that the performance improvement brought by the proposed strategies is statistically significant rather than accidental results caused by random iteration.

### 4.2. CEC2020 High-Dimensional Test Functions

**Figure 4:**
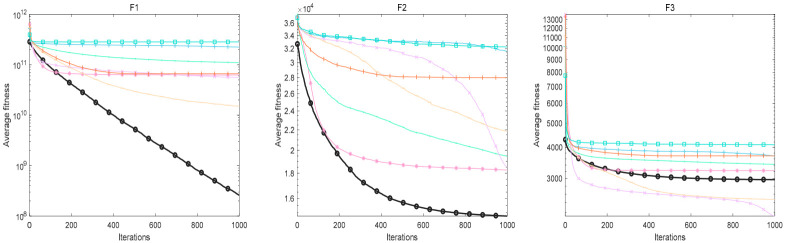

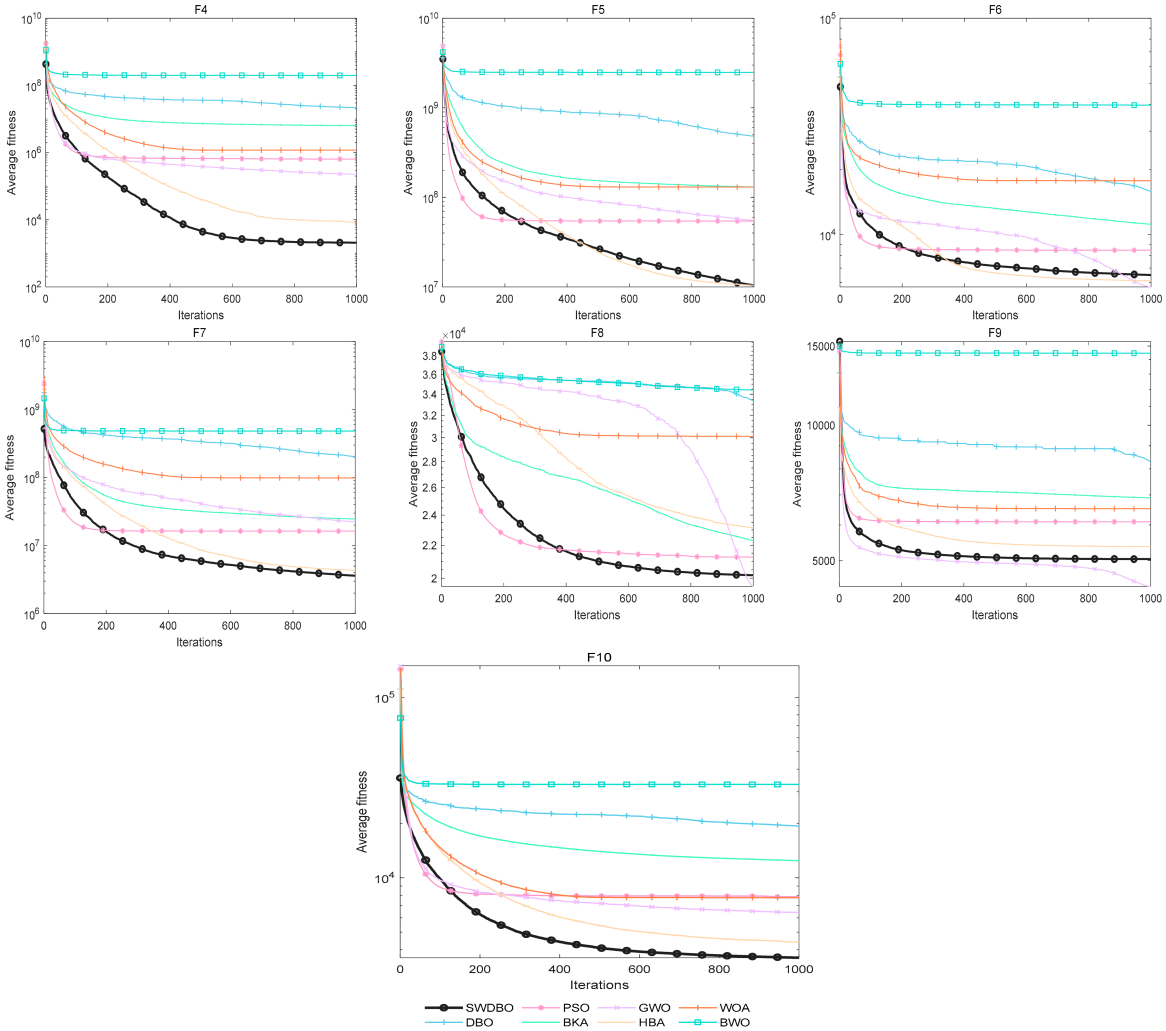
Comparison of average convergence curves of CEC2020 test set algorithms.

**Figure 5:**
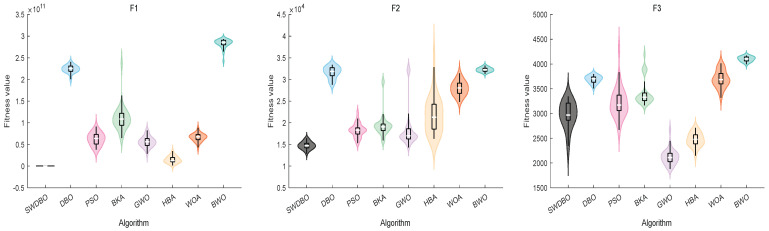

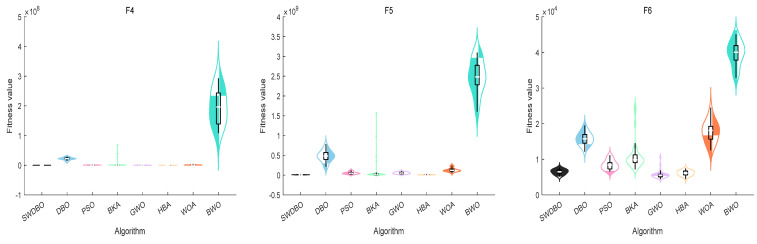

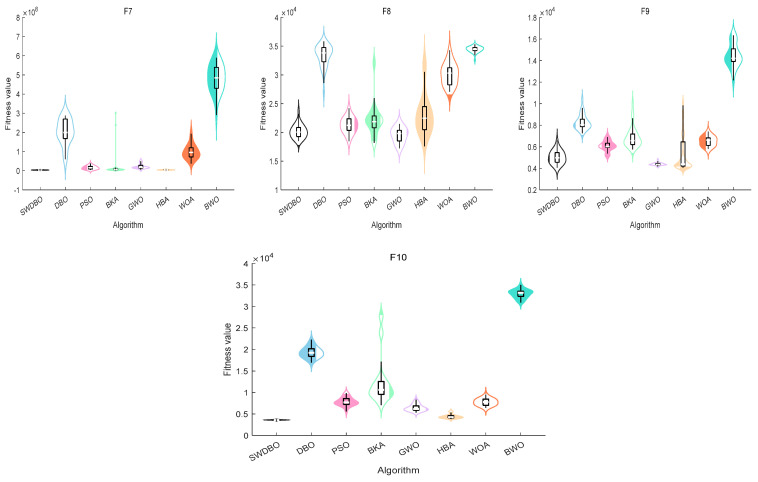
Comparison of violin plots of CEC2020 test set algorithm data.

**Figure 6:**
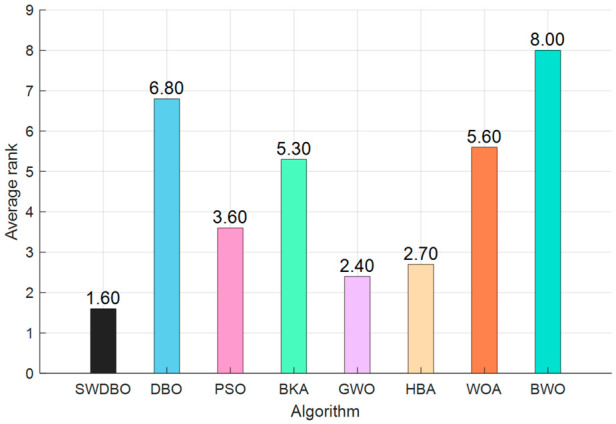
Comparison of average ranking of 8 algorithms in the CEC2020 test set.

CEC2020 includes 10 fully composite test functions. All functions are processed by wide-range coordinate offset and nonlinear rotation. They have dense local optima and more complex surfaces than CEC2017. The 100-dimensional setting further aggravates the curse of dimensionality and puts forward strict requirements on population diversity maintenance and global escape capability. CEC2020 is a well-recognized high-standard benchmark in high-dimensional optimization, which can fully verify the adaptability of improved algorithms to extremely complex search spaces. Table 3 lists the optimization results of the SWDBO and comparative algorithms on CEC2020 test functions. Table 4 presents the Wilcoxon rank-sum test results. Figure 4 shows the average convergence curves. Figure 5 displays the violin plots. Figure 6 illustrates the average ranking of eight algorithms.

In all 10 high-dimensional test problems of CEC2020, SWDBO ranks first in best value and mean value on 9 test functions. Only one function has slightly worse results than BKA. The fixed population proportion of original DBO leads to rapid individual homogenization and loss of population diversity in later iterations of high-dimensional optimization. Both global exploration and local exploitation fall into stagnation, resulting in large optimization errors. Other comparative algorithms are limited by single search modes and easily trapped into local optima on highly distorted composite surfaces. Three improvement strategies of the SWDBO make up for the above defects. The adaptive population ratio delays individual homogenization from the perspective of population structure. The whale bubble-net mechanism improves the efficiency of local refined search. The seagull operator combined with Lévy flight endows individuals with long-distance jump capability to escape from dense local optima. Thus, SWDBO maintains top-level optimization accuracy on the high-difficulty CEC2020 benchmark. Its standard deviations on all test functions are significantly lower than other algorithms, which means small fluctuations in multiple runs and excellent robustness.

All functions in CEC2020 are highly distorted composite high-dimensional problems with explosively numerous local optima, which impose stringent requirements on the algorithms’ capacity to balance exploration and exploitation. Different from the DBO, L-SHADE, BWO and other competitors that suffer from unbalanced search and premature convergence plateaus in middle/late iterations, the three integrated strategies of the SWDBO constitute a complete temporal regulation system to fundamentally optimize convergence evolution. The adaptive allocation strategy balances search resource distribution at the population level to avoid insufficient global search in high-dimensional spaces. The WOA operator embedded in rolling beetles shrinks the search step near optimal regions to continuously pursue lower fitness values. The Lévy long-jump mechanism equipped on thief beetles provides continuous cross-domain search ability to escape clusters of local optima. Benefiting from the coordination of three modules, the SWDBO maintains stable population diversity on all highly distorted CEC2020 landscapes, and its convergence curves descend steadily without obvious flat plateaus. The SWDBO achieves more balanced and long-lasting synergy between global exploration and local exploitation compared with other algorithms, which fully verifies the unique superiority of the multi-strategy integrated framework under extreme high-dimensional composite optimization scenarios.

According to convergence curves, the fitness of all comparative algorithms remains unchanged in later iterations, indicating deep premature convergence. The curve of the SWDBO keeps declining slowly without obvious plateau stages. In terms of CPU running time, the SWDBO has the same time magnitude as the original DBO. As analyzed in complexity analysis, the improvement strategies only replace iteration operators without introducing nested loops or high computational load. The optimization performance is greatly enhanced without a sharp increase in computational cost, which verifies the high efficiency of the SWDBO. The cooperative effect of three strategies guarantees excellent convergence performance. The whale bubble-net strategy helps the algorithm approach optimal regions quickly. The seagull operator maintains the exploration capability of the population. The adaptive ratio strategy balances global and local search in the whole iteration process. The Wilcoxon rank-sum test further verifies the significant differences between the SWDBO and other algorithms. More than 90% of the test functions show statistically significant performance differences, which proves the effectiveness of the proposed improvement strategies.

### 4.3. Experimental Results on Three Constrained Engineering Optimization Problems

Three classic constrained engineering optimization problems are selected, including three-bar truss lightweight design, ten-bar truss lightweight design and cantilever beam size optimization. All problems take the minimum structural weight or volume as the optimization objective, and are subject to multiple constraints such as stress, displacement and size. The search spaces contain a large number of infeasible regions, which are more difficult to solve than unconstrained CEC function optimization. These problems can effectively verify the application value of the algorithm in practical industrial scenarios. All engineering problems adopt inequality constraints. The penalty function method is used to convert constrained optimization problems into unconstrained problems.

(1) Three-bar truss design problem

The design problem of a three-bar truss is a classic structural optimization problem, whose objective is to minimize the volume (or weight) of the truss structure while satisfying stress constraints. The optimization of a three-bar truss is a bivariate optimization problem with multiple constraints, where variables are coupled and the feasible domain is narrow. Most optimization algorithms tend to fall into suboptimal solutions at the boundary of the feasible domain. In order to minimize the volume of the three-bar truss, constraints need to be applied to each truss component, including three types of constraints, namely, buckling constraints, deflection constraints, and stress constraints. This problem has two core parameters: *A*_1_ and *A*_2_. The objective function and constraint conditions are expressed as follows. The optimization variables are defined as $x=[x_{1},x_{2}]=[A_{1},A_{2}]$.

Objective function:

(21) $$Min;f\left(\vec{x}\right)=\left(2\sqrt{2},x_{1}+x_{2}\right)\cdot l$$

Constraints:

(22) $$g_{1}(\vec{x})=\frac{\sqrt{2},x_{1}+x_{2}}{\sqrt{2x_{1}^{2}+2x_{1}x_{2}}},P-\sigma \leq 0$$

(23) $$g_{2}(\vec{x})=\frac{x_{2}}{\sqrt{2x_{1}^{2}+2x_{1}x_{2}}},P-\sigma \leq 0$$

(24) $$g_{3}(\vec{x})=\frac{1}{\sqrt{2x_{2}+x_{1}}},P-\sigma \leq 0$$

Variable range: $0\leq x_{1},x_{2}\leq 1$. Constant parameters: l=100 cm, $P=2{\;\mathrm{kg}/\mathrm{cm}}^{2}$, $\sigma =2\;{\mathrm{kg}/\mathrm{cm}}^{2}$.

**Figure 7:**
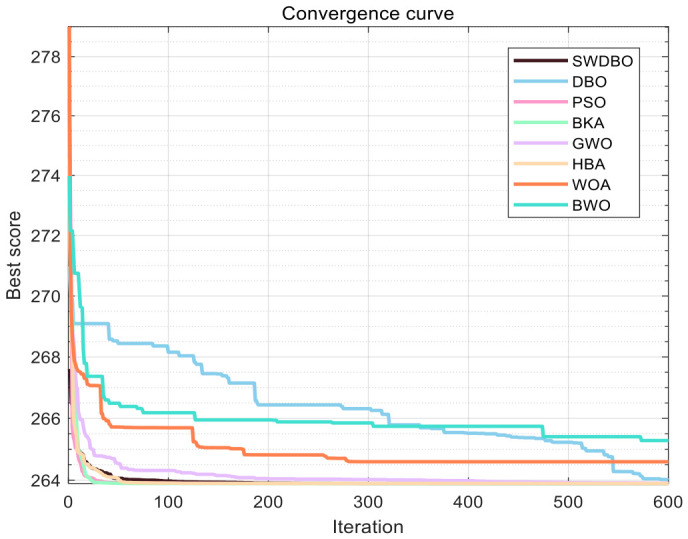
Comparison of average convergence curves for three-bar truss design.

The constraints require that the stress of each rod does not exceed the allowable stress of materials. Figure 7 shows the average convergence curves. Table 5 lists the optimization results.

Table 5 records six evaluation metrics (best, worst, standard deviation, mean, median and average runtime) of the SWDBO and seven peer algorithms including the DBO, PSO, BKA, GWO, HBA, WOA, and BWO over 30 independent runs for the constrained lightweight optimization of the three-bar truss. This benchmark is a typical two-dimensional optimization problem with tightly coupled stress constraints and narrow feasible regions. Most metaheuristics easily converge to suboptimal local solutions on constraint boundaries, which makes it a standard test to evaluate the constraint search robustness and global optimization capacity of optimizers.

In terms of the best objective value (structural volume), the SWDBO achieves the global optimum of 2.64 × 10^2^, tied with GWO and HBA, and outperforms the DBO, PSO, WOA and BWO. Although the WOA also obtains the optimal value in the single trial, its worst result rises to 2.66 × 10^2^, while the BWO reaches 2.67 × 10^2^, indicating they cannot stably reproduce the optimal structural configuration across repeated simulations. The vanilla DBO shares the same best value as the SWDBO, yet its standard deviation is 1.41 × 10^−1^, nearly 100 times larger than the SWDBO (1.43 × 10^−3^). This phenomenon originates from the fixed population proportion of the original DBO, which causes rapid population homogenization in late iterations and frequent deviation from optimal cross-sectional combinations. The BKA outputs meaningless negative objective values throughout all trials, proving it lacks constraint boundary recognition ability and constantly generates infeasible structural schemes exceeding allowable material stress, which disqualifies it for mechanical constrained optimization.

For robustness indicators (worst value, standard deviation, median), the SWDBO exhibits a dominant performance. Its worst result still remains 2.64 × 10^2^ without degraded suboptimal designs, and the standard deviation of 1.43 × 10^−2^ is the minimum among all competitors, which demonstrates that the truss volume obtained under different random initializations barely fluctuates, fully satisfying the repeatability requirement of industrial structural simulation. The median value of the SWDBO equals its global optimum, meaning more than half of independent simulations accurately capture the optimal rod cross-section ratio. In contrast, the medians of the DBO, PSO and GWO are all larger than their respective best values, as most iterations are trapped in local lightweight suboptimal points and fail to escape local constraint traps.

(2) Ten-bar truss design problem

The ten-bar truss design is a benchmark problem in structural optimization with higher complexity. It is a ten-variable high-dimensional constrained optimization problem with complex variable coupling and fragmented feasible regions. Traditional algorithms are easily trapped into local optima in fragmented feasible subspaces and obtain trusses with excessive weight. The optimization objective is to minimize the truss weight under frequency constraints. Related parameters: l=914.4 cm, P=45.400 kg, material density $\rho =2770\;{\mathrm{kg}/m}^{3}$, elastic modulus E=69.8 GPa. The lower bound of all cross-sectional areas is $0.65\;{\mathrm{cm}}^{2}$. Concentrated masses are added to nodes 2, 3, 4 and 5. Frequency constraints: $\omega_{1}\geq 7\;\mathrm{Hz}$, $\omega_{2}\geq 15\;\mathrm{Hz}$, $\omega_{3}\geq 20\;\mathrm{Hz}$.

Objective function:

(25) $$f\left(\vec{x}\right)=\sum_{i=1}^{10}L_{i}\left(x_{i}\right)\rho A_{i}$$

Constraints:

(26) $$g_{1}(\vec{x})=\frac{7}{\omega_{1}(\vec{x})}-1\leq 0$$

(27) $$g_{2}(\vec{x})=\frac{15}{\omega_{2}(\vec{x})}-1\leq 0$$

(28) $$g_{3}(\vec{x})=\frac{20}{\omega_{3}(\vec{x})}-1\leq 0$$

where $6.45\times 10^{-5}\leq A_{i}\leq 5\times 10^{-3},i=1,2,\ldots ,10$.

**Figure 8:**
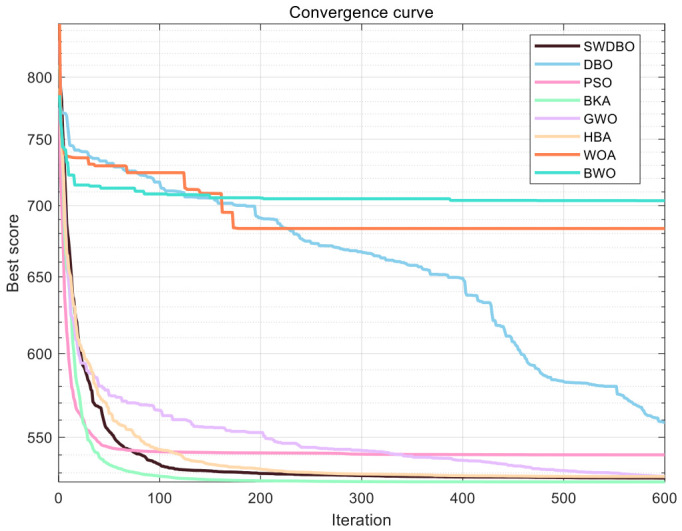
Comparison of average convergence curves for ten-bar truss design.

Variable bounds: $\vec{x}=A_{1},A_{2},\ldots ,A_{10},\rho =2770$. The comparison of the average convergence curves of the ten-bar truss design is shown in Figure 8. The comparison data of the design optimization of the ten-bar truss are shown in Table 6.

Table 6 presents statistical metrics of 30 repeated simulations for ten-bar truss lightweight design subject to multi-order frequency limits. This case is a high-dimensional constrained engineering problem with ten strongly coupled design variables and fragmented feasible regions scattered around constraint boundaries, containing numerous discrete local optima. It imposes strict requirements on the optimizer’s ability to maintain population diversity, search under multiple coupled constraints and jump out of dense local traps. Comprehensive comparison of eight algorithms fully validates the unique superiority of the SWDBO’s multi-strategy framework in high-dimensional industrial optimization. In terms of the minimal structural mass, the SWDBO reaches 5.25 × 10^2^ and ranks equal to the BKA. Nevertheless, the BKA suffers critical drawbacks: it generates massive infeasible negative objective values violating frequency constraints across most trials, and only accidentally finds a feasible optimal solution once, which cannot be applied to repeated engineering design. The GWO and HBA also obtain the same best value, yet their worst solutions rise to 5.32 × 10^2^ and 5.35 × 10^2^ respectively, with much higher mean mass than the SWDBO. The vanilla DBO’s optimal weight is 5.36 × 10^2^, 2.09% heavier than the SWDBO. This reveals that the fixed sub-population ratio of the original DBO fails to adapt to ten-dimensional search spaces, lacking sufficient local exploitation agents in late iterations to discover lighter rod cross-section combinations. The WOA and BWO show the worst performance, with optimal masses of 6.14 × 10^2^ and 6.36 × 10^2^, as single-mechanism metaheuristics suffer severe premature convergence under high-dimensional multi-frequency constraints.

Huge gaps exist in robustness metrics. The SWDBO’s worst value of 5.32 × 10^2^ is the minimum among all feasible algorithms, with a standard deviation of only 2.40, far smaller than the DBO (17.8), PSO (16.2), GWO (2.63) and HBA (3.08). Although the BKA has slightly lower standard deviation, its massive infeasible results render the statistic meaningless. The average structural mass of the SWDBO is 5.27 × 10^2^, the lowest of all feasible optimizers, achieving a 5.72% weight reduction compared with the DBO and 2.41% lighter than PSO. In batch engineering simulations, the SWDBO consistently outputs lightweight truss schemes and significantly cuts steel manufacturing costs. Its median value of 5.27 × 10^2^ is extremely close to the global optimum, indicating most simulation runs converge to the optimal neighborhood. In contrast, the DBO’s median reaches 5.53 × 10^2^, demonstrating most iterations are trapped in heavy local configurations with weak multi-constraint boundary search capacity.

(3) Welded beam design problem

The optimization objective of cantilever beam design is to minimize the beam weight under bending stress constraints. The problem takes beam width, beam height and beam length as optimization variables, which are constrained by bending stress and deflection. Traditional algorithms are easily restricted by constraint boundaries. Three continuous design variables are adopted: beam width b ($X_{1}$), beam height h ($X_{2}$) and beam length L ($X_{3}$). Variable range: $0.1\leq X_{1}\leq 10$, $0.1\leq X_{2}\leq 10$, $1\leq X_{3}\leq 20$ (unit: inch), which conforms to the actual size range of cantilever beams.

Objective function. The objective function is to minimize the weight of the cantilever beam, which is made of steel with a density of $\rho =0.283\;{\mathrm{lb}/\mathrm{in}}^{3}$. The expression of the objective function is

(29) $$\min\;f(X)=\rho \times X_{1}\times X_{2}\times X_{3}$$

Constraints. The core constraint of cantilever beam design is bending stress constraint, which must not exceed the maximum allowable value and must also meet geometric dimension constraints, as follows:

(30) $$\left\{\begin{array}{l} \sigma (X)\leq \sigma_{\max}\\ 0.1-X_{1}\leq 0\\ 0.1-X_{2}\leq 0\\ 1-X_{3}\leq 0 \end{array}\right.$$

In the formula, $\sigma \left(X\right)$ is the bending stress of the cantilever beam, and its calculation formula is $\sigma \left(X\right)=\frac{6PL}{X_{1}X_{2}^{2}}$. *P* = 1000 lb (applied load). $\sigma_{\max}$ = 30,000 psi (maximum allowable bending stress). The remaining constraints are geometric size constraints to ensure that the design variables meet the actual manufacturing requirements.

**Figure 9:**
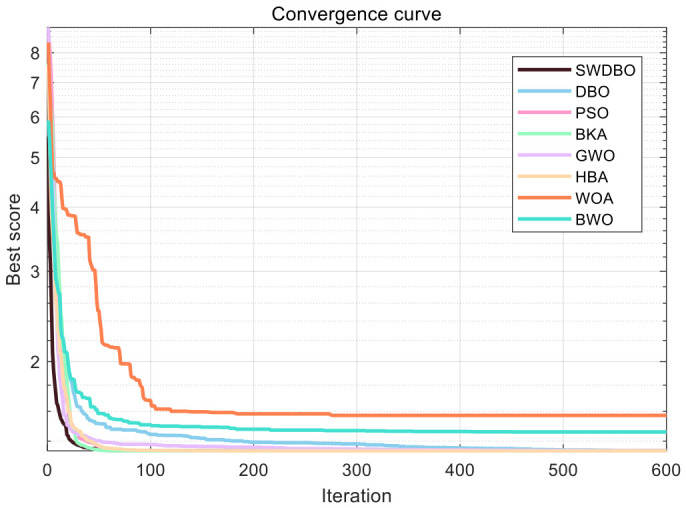
Comparison of average convergence curves for optimized welding beam design.

The comparison of average convergence curves for welding beam design optimization is shown in Figure 9. The comparison data of optimized welding beam design are shown in Table 7.

Table 7 summarizes all statistical indicators of 30 independent simulations for cantilever beam minimum-weight design. This three-dimensional engineering problem subjects to upper bending stress limits and geometric dimension boundaries, characterized by wide variable ranges and highly nonlinear stress constraints with dense local optima. It effectively evaluates the integrated local exploitation and global escape capacity of optimizers under medium-dimensional nonlinear mechanical limits. In terms of the minimal beam mass, SWDBO achieves the optimal value of 1.34 × 10^0^, equal to the DBO, PSO, BKA, GWO and HBA. The WOA and BWO only reach 1.38 × 10^0^ with much lower material utilization efficiency. Although the vanilla DBO occasionally finds the lightest configuration in a single run, its standard deviation is 2.46 × 10^−4^, over 35 times larger than the SWDBO (6.89 × 10^−6^), which reveals the terrible iteration stability of the original DBO, and most random initializations generate overweight beam structures. The BKA also obtains the best value occasionally yet frequently outputs designs exceeding allowable bending stress, leading to low engineering reliability. The SWDBO’s standard deviation of merely 6.89 × 10^−6^ ranks the minimum across all competitors, and the beam weight from 30 trials barely fluctuates, fully meeting repeatability requirements of mechanical batch design.

The worst value index directly reflects the SWDBO’s strong anti-premature performance: its worst solution remains 1.34 × 10^0^ without any overweight inferior design. In contrast, the WOA’s worst mass reaches 1.70 × 10^0^ and the BWO hits 1.50 × 10^2^, meaning many iterations converge to thick heavy beam configurations near stress boundaries and cause severe material waste. The average mass of the SWDBO is identical to its global optimum at 1.34 × 10^0^. While the DBO and PSO share the same mean value, their standard deviations are orders of magnitude higher, showing huge gaps in random simulation stability. The median of the SWDBO equals its best result, proving all repeated simulations steadily locate the minimal weight dimension combination; the medians of other algorithms are no less than the optimum, with most iterations trapped in suboptimal local sizing schemes.

In computational performance, the SWDBO takes an average runtime of 0.198 s, slower than the DBO, PSO, GWO and HBA but much faster than the BKA and BWO. The three-dimensional variable scene brings negligible computing burden to the hybrid operators without iteration explosion, supporting rapid batch parameter optimization for lightweight mechanical component design. The comprehensive results derived from three constrained structural optimization benchmarks lead to consistent conclusions regarding the performance of the SWDBO. The proposed multi-strategy framework achieves the lightest structural weight for all three engineering cases, which fully validates the effectiveness of the integrated improvement scheme. Compared with the vanilla DBO, the SWDBO exhibits remarkable performance gains, which can be attributed to the synergistic interplay among the adaptive population allocation strategy, WOA bubble-net local search mechanism and modified seagull/Lévy jump operator. The convergence curves demonstrate that the SWDBO undergoes rapid fitness reduction at early iterations and owns the fastest convergence rate among all competitors. This prominent fast convergence property originates from the bubble-net search embedded in rolling beetles, which enables precise exploitation around promising optimal candidates and accelerates the descent of objective values. Over 30 repeated simulations, the SWDBO maintains the smallest standard deviation of fitness metrics, which confirms its superior robustness against random initialization. The adaptive population ratio dynamically reorganizes the proportion of search agents at different evolutionary phases, mitigating the performance volatility induced by random population initialization. When tackling engineering tasks with highly nonlinear inequality constraints, the SWDBO cooperates with the penalty function approach to efficiently probe constraint boundaries and locate high-quality feasible solutions that fully satisfy all mechanical limit conditions. Comparative experiments against the DBO, PSO, BKA, GWO, HBA, WOA and BWO reveal the comprehensive superiority of the SWDBO for constrained engineering optimization. Such competitive edges become especially prominent for the high-dimensional ten-bar truss design problem, which further demonstrates the great application potential of the proposed algorithm in complex high-dimensional engineering optimization tasks.

## 5. Conclusions

Aiming at the defects of basic DBO in solving high-dimensional complex optimization problems, including slow convergence, local optimum trapping and decreased population diversity in later iterations, a multi-strategy improved Dung Beetle Optimizer (SWDBO) is proposed in this paper. Three main improvements are summarized as follows. First, an adaptive population ratio strategy is designed to dynamically adjust the quantity of different types of dung beetles and enhance population diversity during iterations. Second, the bubble-net predation strategy of the WOA is integrated into the local search of rolling beetles to strengthen local search ability and accelerate convergence speed. Third, the spiral attack strategy of the improved Seagull Optimization Algorithm, together with adaptive inertia weight, nonlinear decreasing factor and Lévy flight strategy, is introduced into the position update of brood beetles and thief beetles to reduce the probability of local optimum trapping. Theoretical complexity analysis shows that the time complexity of the SWDBO is consistent with the original DBO. The improvement strategies enhance optimization performance without obvious increase in computational burden. Comparative experiments on high-dimensional benchmark functions from CEC2017 and CEC2020 are carried out. The SWDBO is compared with the DBO, PSO, BKA, GWO, HBA, WOA and BWO. The experimental results demonstrate that the SWDBO is superior to comparative algorithms in convergence accuracy, convergence speed and robustness. The Wilcoxon rank-sum test verifies the statistical significance of performance improvement. The SWDBO is applied to three engineering optimization problems, including three-bar truss design, ten-bar truss design and cantilever beam design. The results fully prove the effectiveness and superiority of the proposed algorithm in practical engineering applications. The SWDBO provides a new effective method for solving high-dimensional optimization and engineering application problems.

Although the proposed SWDBO achieves outstanding optimization performance on 100-dimensional continuous numerical benchmarks and three constrained structural engineering problems, several inherent limitations and expansion bottlenecks remain to be addressed. First, the stacked three sets of improved operators slightly increase the computational cost of single individual iteration when dealing with ultra-large populations containing hundreds of thousands of agents, which restricts its deployment on embedded devices with extremely limited computing resources. Second, the hyperparameters embedded in the SWDBO, such as adaptive coefficients, Lévy flight β and WOA safety threshold, perform stably under conventional 100-dimensional tasks but require manual fine-tuning for ultra-high-dimensional problems with over 500 variables, resulting in certain parameter dependency. Third, the update formulas of the SWDBO are exclusively constructed for continuous variables without customized iterative rules for discrete search spaces; thus the algorithm exhibits insufficient adaptability when directly applied to discrete optimization tasks such as job-shop scheduling and route planning, as well as multi-objective optimization problems. The SWDBO maintains stable optimization performance on 100-dimensional CEC2017/CEC2020 composite functions, whereas baseline algorithms (DBO, PSO, WOA) show obvious accuracy collapse with rising dimensions. The SWDBO can be seamlessly transplanted to different constrained structural optimization tasks (2D three-bar truss, 10D ten-bar truss, 3D cantilever beam), verifying strong cross-task scalability of the proposed framework.

Future research can be carried out from the following aspects. First, extend the SWDBO to multi-objective optimization, design a multi-objective improved Dung Beetle Optimizer and apply it to more complex engineering scenarios. Second, combine machine learning technology with SWDBO, and dynamically adjust algorithm parameters via adaptive learning strategies to further improve the self-adaptability of the algorithm. Third, apply the SWDBO to a wider range of practical applications, such as hyperparameter optimization of deep learning models, unmanned aerial vehicle path planning and power system scheduling. The improved SWDBO can be applied to parameter optimization of new energy units, multi-constraint path planning of unmanned aerial vehicles, multi-objective lightweight design of complex equipment and other difficult engineering optimization problems. The multi-objective version of the SWDBO can be further improved to solve high-dimensional multi-objective optimization problems.

## Figures and Tables

**Table 1:** Results of different swarm intelligence algorithms of CEC2017.

Function	Metric	SWDBO	DBO	PSO	BKA	GWO	HBA	WOA	BWO
F1	Best	1.46 × 10^8^	1.97 × 10^11^	3.48 × 10^10^	8.37 × 10^10^	2.94 × 10^10^	3.58 × 10^9^	4.86 × 10^10^	2.58 × 10^11^
F1	Std	1.98 × 10^8^	1.08 × 10^10^	1.04 × 10^10^	4.38 × 10^10^	1.03 × 10^10^	6.09 × 10^9^	8.98 × 10^9^	7.31 × 10^9^
F1	Avg	3.04 × 10^8^	2.23 × 10^11^	5.66 × 10^10^	1.20 × 10^11^	5.03 × 10^10^	1.24 × 10^10^	6.63 × 10^10^	2.85 × 10^11^
F3	Best	2.90 × 10^5^	3.32 × 10^5^	3.39 × 10^5^	1.86 × 10^5^	3.45 × 10^5^	2.89 × 10^5^	6.94 × 10^5^	3.69 × 10^5^
F3	Std	1.85 × 10^4^	9.10 × 10^4^	1.03 × 10^5^	6.80 × 10^4^	6.15 × 10^4^	3.48 × 10^4^	1.14 × 10^5^	1.65 × 10^8^
F3	Avg	3.29 × 10^5^	4.18 × 10^5^	5.03 × 10^5^	2.52 × 10^5^	4.44 × 10^5^	3.35 × 10^5^	9.23 × 10^5^	3.25 × 10^7^
F4	Best	7.57 × 10^2^	4.32 × 10^4^	3.75 × 10^3^	5.49 × 10^3^	3.21 × 10^3^	1.18 × 10^3^	8.04 × 10^3^	1.14 × 10^5^
F4	Std	7.48 × 10^1^	6.30 × 10^3^	2.84 × 10^3^	1.84 × 10^4^	2.28 × 10^3^	2.85 × 10^2^	2.33 × 10^3^	8.31 × 10^3^
F4	Avg	9.18 × 10^2^	5.78 × 10^4^	7.76 × 10^3^	1.68 × 10^4^	6.02 × 10^3^	1.58 × 10^3^	1.17 × 10^4^	1.36 × 10^5^
F5	Best	1.18 × 10^3^	1.83 × 10^3^	1.29 × 10^3^	1.27 × 10^3^	1.07 × 10^3^	1.06 × 10^3^	1.65 × 10^3^	2.08 × 10^3^
F5	Std	4.75 × 10^1^	5.38 × 10^1^	1.06 × 10^2^	1.77 × 10^2^	1.13 × 10^2^	8.22 × 10^1^	1.73 × 10^2^	3.00 × 10^1^
F5	Avg	1.37 × 10^3^	1.94 × 10^3^	1.50 × 10^3^	1.48 × 10^3^	1.22 × 10^3^	1.21 × 10^3^	1.89 × 10^3^	2.14 × 10^3^
F6	Best	6.53 × 10^2^	6.88 × 10^2^	6.52 × 10^2^	6.70 × 10^2^	6.31 × 10^2^	6.40 × 10^2^	6.86 × 10^2^	7.11 × 10^2^
F6	Std	3.61 × 10^0^	4.84 × 10^0^	5.94 × 10^0^	1.19 × 10^1^	5.59 × 10^0^	5.44 × 10^0^	1.06 × 10^1^	1.52 × 10^0^
F6	Avg	6.62 × 10^2^	6.98 × 10^2^	6.69 × 10^2^	6.80 × 10^2^	6.44 × 10^2^	6.48 × 10^2^	7.02 × 10^2^	7.16 × 10^2^
F7	Best	2.48 × 10^3^	3.44 × 10^3^	2.61 × 10^3^	3.12 × 10^3^	1.86 × 10^3^	1.90 × 10^3^	3.46 × 10^3^	3.98 × 10^3^
F7	Std	2.71 × 10^2^	9.65 × 10^1^	2.69 × 10^2^	1.89 × 10^2^	1.56 × 10^2^	2.44 × 10^2^	1.73 × 10^2^	4.41 × 10^1^
F7	Avg	2.98 × 10^3^	3.68 × 10^3^	3.17 × 10^3^	3.38 × 10^3^	2.16 × 10^3^	2.49 × 10^3^	3.72 × 10^3^	4.11 × 10^3^
F8	Best	1.58 × 10^3^	2.23 × 10^3^	1.70 × 10^3^	1.76 × 10^3^	1.34 × 10^3^	1.35 × 10^3^	2.09 × 10^3^	2.53 × 10^3^
F8	Std	7.85 × 10^1^	6.07 × 10^1^	1.04 × 10^2^	2.15 × 10^2^	1.05 × 10^2^	7.42 × 10^1^	1.49 × 10^2^	3.89 × 10^1^
F8	Avg	1.79 × 10^3^	2.33 × 10^3^	1.87 × 10^3^	1.97 × 10^3^	1.55 × 10^3^	1.52 × 10^3^	2.28 × 10^3^	2.65 × 10^3^
F9	Best	2.40 × 10^4^	6.60 × 10^4^	2.34 × 10^4^	2.72 × 10^4^	2.16 × 10^4^	2.26 × 10^4^	4.54 × 10^4^	7.30 × 10^4^
F9	Std	6.70 × 10^2^	5.50 × 10^3^	5.72 × 10^3^	1.29 × 10^4^	1.28 × 10^4^	1.09 × 10^4^	1.76 × 10^4^	2.14 × 10^3^
F9	Avg	2.50 × 10^4^	7.62 × 10^4^	3.22 × 10^4^	3.61 × 10^4^	3.95 × 10^4^	3.45 × 10^4^	6.94 × 10^4^	7.85 × 10^4^
F10	Best	1.35 × 10^4^	1.97 × 10^4^	1.38 × 10^4^	1.61 × 10^4^	1.30 × 10^4^	1.61 × 10^4^	2.37 × 10^4^	3.07 × 10^4^
F10	Std	1.72 × 10^3^	2.46 × 10^3^	1.52 × 10^3^	3.64 × 10^3^	4.05 × 10^3^	3.15 × 10^3^	1.85 × 10^3^	4.99 × 10^2^
F10	Avg	1.59 × 10^4^	3.10 × 10^4^	1.77 × 10^4^	1.92 × 10^4^	1.77 × 10^4^	2.06 × 10^4^	2.80 × 10^4^	3.23 × 10^4^
F11	Best	5.45 × 10^4^	1.47 × 10^5^	5.09 × 10^4^	3.76 × 10^4^	8.73 × 10^4^	4.57 × 10^4^	1.63 × 10^5^	2.11 × 10^5^
F11	Std	1.87 × 10^4^	1.87 × 10^4^	4.35 × 10^4^	2.68 × 10^4^	1.85 × 10^4^	1.23 × 10^4^	1.08 × 10^5^	1.81 × 10^5^
F11	Avg	9.62 × 10^4^	1.90 × 10^5^	9.90 × 10^4^	7.21 × 10^4^	1.18 × 10^5^	6.67 × 10^4^	3.25 × 10^5^	4.27 × 10^5^
F12	Best	2.16 × 10^7^	1.08 × 10^11^	2.22 × 10^9^	5.78 × 10^9^	1.69 × 10^9^	8.01 × 10^7^	9.19 × 10^9^	2.03 × 10^11^
F12	Std	4.07 × 10^7^	1.23 × 10^10^	6.76 × 10^9^	3.61 × 10^10^	4.93 × 10^9^	3.60 × 10^8^	3.43 × 10^9^	1.19 × 10^10^
F12	Avg	7.76 × 10^7^	1.30 × 10^11^	1.20 × 10^10^	3.28 × 10^10^	1.14 × 10^10^	2.67 × 10^8^	1.36 × 10^10^	2.34 × 10^11^
F13	Best	2.42 × 10^4^	2.39 × 10^10^	1.28 × 10^7^	7.20 × 10^6^	2.96 × 10^7^	3.71 × 10^4^	2.10 × 10^8^	4.37 × 10^10^
F13	Std	3.11 × 10^4^	3.51 × 10^9^	1.11 × 10^9^	1.10 × 10^10^	9.82 × 10^8^	2.30 × 10^4^	3.77 × 10^8^	5.00 × 10^9^
F13	Avg	6.35 × 10^4^	3.07 × 10^10^	7.20 × 10^8^	5.75 × 10^9^	1.35 × 10^9^	6.98 × 10^4^	6.40 × 10^8^	5.57 × 10^10^
F14	Best	5.23 × 10^5^	1.35 × 10^7^	8.74 × 10^5^	4.84 × 10^5^	1.90 × 10^6^	3.48 × 10^5^	3.67 × 10^6^	1.09 × 10^8^
F14	Std	3.33 × 10^5^	6.15 × 10^6^	2.79 × 10^6^	1.09 × 10^7^	4.28 × 10^6^	5.24 × 10^5^	8.07 × 10^6^	1.43 × 10^8^
F14	Avg	9.56 × 10^5^	2.42 × 10^7^	4.09 × 10^6^	5.24 × 10^6^	7.00 × 10^6^	1.16 × 10^6^	1.63 × 10^7^	3.00 × 10^8^
F15	Best	4.53 × 10^3^	6.89 × 10^9^	1.67 × 10^4^	2.97 × 10^5^	1.63 × 10^6^	8.28 × 10^3^	1.91 × 10^7^	2.43 × 10^10^
F15	Std	1.57 × 10^4^	1.83 × 10^9^	8.93 × 10^8^	7.72 × 10^9^	5.16 × 10^8^	1.31 × 10^4^	7.09 × 10^7^	3.60 × 10^9^
F15	Avg	3.25 × 10^4^	1.04 × 10^10^	4.32 × 10^8^	3.79 × 10^9^	3.17 × 10^8^	2.27 × 10^4^	9.65 × 10^7^	3.30 × 10^10^
F16	Best	3.92 × 10^3^	1.25 × 10^4^	5.80 × 10^3^	6.73 × 10^3^	5.29 × 10^3^	4.66 × 10^3^	1.12 × 10^4^	2.58 × 10^4^
F16	Std	8.37 × 10^2^	1.46 × 10^3^	7.57 × 10^2^	2.47 × 10^3^	7.17 × 10^2^	7.06 × 10^2^	2.18 × 10^3^	1.73 × 10^3^
F16	Avg	5.89 × 10^3^	1.56 × 10^4^	7.54 × 10^3^	9.28 × 10^3^	6.29 × 10^3^	5.97 × 10^3^	1.50 × 10^4^	2.99 × 10^4^
F17	Best	4.39 × 10^3^	1.15 × 10^4^	4.91 × 10^3^	4.62 × 10^3^	3.90 × 10^3^	4.33 × 10^3^	8.22 × 10^3^	1.41 × 10^6^
F17	Std	5.62 × 10^2^	9.93 × 10^4^	8.47 × 10^2^	5.60 × 10^5^	1.27 × 10^3^	7.48 × 10^2^	1.42 × 10^4^	2.78 × 10^7^
F17	Avg	5.73 × 10^3^	8.91 × 10^4^	6.52 × 10^3^	1.46 × 10^5^	5.66 × 10^3^	5.76 × 10^3^	1.46 × 10^4^	4.14 × 10^7^
F18	Best	6.37 × 10^5^	1.18 × 10^7^	7.52 × 10^5^	4.95 × 10^5^	2.23 × 10^6^	5.99 × 10^5^	1.32 × 10^6^	1.13 × 10^8^
F18	Std	1.18 × 10^6^	1.77 × 10^7^	3.90 × 10^6^	3.44 × 10^7^	3.46 × 10^6^	1.33 × 10^6^	4.56 × 10^6^	2.12 × 10^8^
F18	Avg	2.57 × 10^6^	3.30 × 10^7^	5.61 × 10^6^	2.00 × 10^7^	7.44 × 10^6^	2.37 × 10^6^	1.25 × 10^7^	6.86 × 10^8^
F19	Best	2.67 × 10^3^	4.27 × 10^9^	2.69 × 10^5^	4.92 × 10^6^	1.79 × 10^7^	2.94 × 10^3^	4.81 × 10^7^	2.69 × 10^10^
F19	Std	7.45 × 10^3^	2.64 × 10^9^	2.48 × 10^8^	4.15 × 10^9^	2.56 × 10^8^	2.32 × 10^5^	1.19 × 10^8^	3.71 × 10^9^
F19	Avg	1.05 × 10^4^	8.55 × 10^9^	1.53 × 10^8^	1.85 × 10^9^	2.24 × 10^8^	5.87 × 10^4^	1.66 × 10^8^	3.37 × 10^10^
F20	Best	4.36 × 10^3^	6.25 × 10^3^	4.65 × 10^3^	4.83 × 10^3^	4.00 × 10^3^	3.53 × 10^3^	6.09 × 10^3^	7.25 × 10^3^
F20	Std	5.84 × 10^2^	4.78 × 10^2^	4.70 × 10^2^	3.08 × 10^2^	7.48 × 10^2^	6.74 × 10^2^	6.16 × 10^2^	3.18 × 10^2^
F20	Avg	5.40 × 10^3^	7.37 × 10^3^	5.72 × 10^3^	5.38 × 10^3^	4.96 × 10^3^	5.28 × 10^3^	6.97 × 10^3^	7.96 × 10^3^
F21	Best	9.27 × 10^3^	1.71 × 10^4^	9.72 × 10^3^	1.27 × 10^4^	7.73 × 10^3^	3.30 × 10^3^	1.47 × 10^4^	2.05 × 10^4^
F21	Std	1.25 × 10^3^	5.68 × 10^2^	1.17 × 10^3^	2.05 × 10^3^	7.59 × 10^2^	1.42 × 10^3^	1.20 × 10^3^	4.80 × 10^2^
F21	Avg	1.16 × 10^4^	1.84 × 10^4^	1.27 × 10^4^	1.49 × 10^4^	9.40 × 10^3^	9.11 × 10^3^	1.78 × 10^4^	2.17 × 10^4^
F22	Best	4.21 × 10^4^	6.05 × 10^4^	4.36 × 10^4^	4.33 × 10^4^	3.90 × 10^4^	4.52 × 10^4^	6.62 × 10^4^	8.83 × 10^4^
F22	Std	6.88 × 10^3^	6.48 × 10^3^	4.43 × 10^3^	9.42 × 10^3^	9.34 × 10^3^	1.10 × 10^4^	4.14 × 10^3^	1.52 × 10^3^
F22	Avg	5.12 × 10^4^	7.88 × 10^4^	5.25 × 10^4^	5.70 × 10^4^	5.00 × 10^4^	5.76 × 10^4^	7.54 × 10^4^	9.05 × 10^4^
F23	Best	8.25 × 10^3^	2.03 × 10^4^	1.43 × 10^4^	1.61 × 10^4^	9.29 × 10^3^	7.91 × 10^3^	1.87 × 10^4^	2.59 × 10^4^
F23	Std	1.62 × 10^3^	7.39 × 10^2^	1.61 × 10^3^	3.16 × 10^3^	7.13 × 10^2^	1.07 × 10^3^	2.02 × 10^3^	1.91 × 10^3^
F23	Avg	1.15 × 10^4^	2.16 × 10^4^	1.73 × 10^4^	2.04 × 10^4^	1.06 × 10^4^	9.61 × 10^3^	2.26 × 10^4^	3.16 × 10^4^
F24	Best	2.86 × 10^3^	2.21 × 10^4^	1.57 × 10^4^	1.92 × 10^4^	1.03 × 10^4^	1.26 × 10^4^	2.06 × 10^4^	2.44 × 10^4^
F24	Std	2.88 × 10^3^	3.28 × 10^2^	1.29 × 10^3^	1.59 × 10^3^	1.03 × 10^3^	1.53 × 10^3^	1.18 × 10^3^	2.69 × 10^2^
F24	Avg	1.73 × 10^4^	2.28 × 10^4^	1.78 × 10^4^	2.06 × 10^4^	1.22 × 10^4^	1.59 × 10^4^	2.29 × 10^4^	2.51 × 10^4^
F25	Best	3.42 × 10^3^	1.74 × 10^4^	5.58 × 10^3^	6.98 × 10^3^	4.79 × 10^3^	3.91 × 10^3^	6.79 × 10^3^	2.97 × 10^4^
F25	Std	7.53 × 10^1^	1.41 × 10^3^	1.59 × 10^3^	5.83 × 10^3^	1.35 × 10^3^	4.19 × 10^2^	6.74 × 10^2^	1.30 × 10^3^
F25	Avg	3.56 × 10^3^	1.94 × 10^4^	7.94 × 10^3^	1.20 × 10^4^	6.81 × 10^3^	4.46 × 10^3^	7.93 × 10^3^	3.25 × 10^4^
F26	Best	4.42 × 10^3^	3.25 × 10^4^	2.32 × 10^4^	2.37 × 10^4^	1.42 × 10^4^	1.11 × 10^4^	2.97 × 10^4^	5.53 × 10^4^
F26	Std	8.45 × 10^3^	2.86 × 10^3^	2.95 × 10^3^	6.50 × 10^3^	1.09 × 10^3^	3.01 × 10^3^	2.86 × 10^3^	1.67 × 10^3^
F26	Avg	1.42 × 10^4^	3.78 × 10^4^	2.88 × 10^4^	3.35 × 10^4^	1.62 × 10^4^	1.53 × 10^4^	3.75 × 10^4^	5.87 × 10^4^
F27	Best	3.48 × 10^3^	6.45 × 10^3^	3.91 × 10^3^	4.35 × 10^3^	3.87 × 10^3^	3.65 × 10^3^	4.81 × 10^3^	1.23 × 10^4^
F27	Std	1.90 × 10^2^	9.72 × 10^2^	4.22 × 10^2^	1.31 × 10^3^	2.25 × 10^2^	2.03 × 10^3^	8.14 × 10^2^	1.21 × 10^3^
F27	Avg	3.73 × 10^3^	8.30 × 10^3^	4.56 × 10^3^	5.88 × 10^3^	4.27 × 10^3^	5.10 × 10^3^	5.85 × 10^3^	1.58 × 10^4^
F28	Best	3.53 × 10^3^	1.73 × 10^4^	7.57 × 10^3^	1.00 × 10^4^	6.96 × 10^3^	4.08 × 10^3^	9.61 × 10^3^	3.06 × 10^4^
F28	Std	6.65 × 10^1^	1.73 × 10^3^	2.40 × 10^3^	3.85 × 10^3^	1.24 × 10^3^	4.18 × 10^2^	9.15 × 10^2^	8.33 × 10^2^
F28	Avg	3.65 × 10^3^	2.17 × 10^4^	1.20 × 10^4^	1.34 × 10^4^	9.75 × 10^3^	4.86 × 10^3^	1.09 × 10^4^	3.28 × 10^4^
F29	Best	5.64 × 10^3^	2.49 × 10^4^	8.62 × 10^3^	9.81 × 10^3^	7.82 × 10^3^	6.05 × 10^3^	1.47 × 10^4^	3.36 × 10^5^
F29	Std	5.30 × 10^2^	2.75 × 10^4^	1.12 × 10^3^	5.07 × 10^4^	8.69 × 10^2^	5.94 × 10^2^	3.18 × 10^3^	1.01 × 10^6^
F29	Avg	7.14 × 10^3^	6.42 × 10^4^	1.02 × 10^4^	2.48 × 10^4^	9.03 × 10^3^	7.58 × 10^3^	1.91 × 10^4^	2.07 × 10^6^
F30	Best	9.06 × 10^4^	1.96 × 10^10^	3.13 × 10^7^	3.17 × 10^7^	9.08 × 10^7^	3.98 × 10^5^	6.28 × 10^8^	2.97 × 10^10^
F30	Std	9.92 × 10^5^	3.16 × 10^9^	9.12 × 10^8^	1.12 × 10^10^	9.69 × 10^8^	8.68 × 10^5^	5.96 × 10^8^	5.23 × 10^9^
F30	Avg	9.60 × 10^5^	2.54 × 10^10^	8.34 × 10^8^	5.91 × 10^9^	1.07 × 10^9^	1.33 × 10^6^	1.57 × 10^9^	5.04 × 10^10^

**Table 2:** Wilcoxon rank-sum test results comparison for CEC2017.

Function	DBO	PSO	BKA	GWO	HBA	WOA	BWO
F1	3.02 × 10^−11^	3.02 × 10^−11^	3.02 × 10^−11^	3.02 × 10^−11^	3.02 × 10^−11^	3.02 × 10^−11^	3.02 × 10^−11^
F3	2.39 × 10^−8^	6.70 × 10^−11^	2.20 × 10^−7^	9.92 × 10^−11^	7.73 × 10^−1^	3.02 × 10^−11^	3.34 × 10^−11^
F4	3.02 × 10^−11^	3.02 × 10^−11^	3.02 × 10^−11^	3.02 × 10^−11^	3.02 × 10^−11^	3.02 × 10^−11^	3.02 × 10^−11^
F5	3.02 × 10^−11^	1.86 × 10^−6^	3.83 × 10^−6^	9.26 × 10^−9^	1.86 × 10^−9^	3.02 × 10^−11^	3.02 × 10^−11^
F6	3.02 × 10^−11^	9.53 × 10^−7^	3.02 × 10^−11^	5.49 × 10^−11^	2.15 × 10^−10^	3.02 × 10^−11^	3.02 × 10^−11^
F7	3.02 × 10^−11^	1.70 × 10^−2^	7.69 × 10^−8^	3.02 × 10^−11^	9.06 × 10^−8^	3.02 × 10^−11^	3.02 × 10^−11^
F8	3.02 × 10^−11^	7.62 × 10^−3^	1.34 × 10^−5^	1.17 × 10^−9^	7.39 × 10^−11^	3.02 × 10^−11^	3.02 × 10^−11^
F9	3.02 × 10^−11^	6.01 × 10^−8^	3.02 × 10^−11^	1.25 × 10^−5^	1.61 × 10^−6^	3.02 × 10^−11^	3.02 × 10^−11^
F10	3.34 × 10^−11^	1.17 × 10^−5^	5.09 × 10^−8^	3.85 × 10^−3^	9.26 × 10^−9^	3.02 × 10^−11^	3.22 × 10^−11^
F11	3.02 × 10^−11^	3.33 × 10^−1^	2.00 × 10^−6^	1.04 × 10^−4^	5.09 × 10^−8^	3.02 × 10^−11^	3.02 × 10^−11^
F12	3.02 × 10^−11^	3.02 × 10^−11^	3.02 × 10^−11^	3.02 × 10^−11^	1.56 × 10^−8^	3.02 × 10^−11^	3.02 × 10^−11^
F13	3.02 × 10^−11^	3.02 × 10^−11^	3.02 × 10^−11^	3.02 × 10^−11^	1.54 × 10^−1^	3.02 × 10^−11^	3.02 × 10^−11^
F14	3.02 × 10^−11^	1.29 × 10^−9^	3.18 × 10^−1^	3.34 × 10^−11^	7.98 × 10^−2^	3.02 × 10^−11^	3.05 × 10^−11^
F15	3.02 × 10^−11^	3.08 × 10^−8^	3.02 × 10^−11^	3.02 × 10^−11^	3.50 × 10^−3^	3.02 × 10^−11^	3.02 × 10^−11^
F16	3.02 × 10^−11^	1.56 × 10^−8^	1.21 × 10^−10^	4.68 × 10^−2^	5.59 × 10^−1^	3.02 × 10^−11^	3.02 × 10^−11^
F17	3.02 × 10^−11^	3.01 × 10^−4^	6.05 × 10^−7^	4.21 × 10^−2^	8.88 × 10^−1^	3.02 × 10^−11^	3.02 × 10^−11^
F18	3.02 × 10^−11^	1.68 × 10^−4^	1.00 × 10^0^	4.18 × 10^−9^	3.79 × 10^−1^	6.72 × 10^−10^	3.08 × 10^−11^
F19	3.02 × 10^−11^	3.02 × 10^−11^	3.02 × 10^−11^	3.02 × 10^−11^	9.63 × 10^−2^	3.02 × 10^−11^	3.02 × 10^−11^
F20	3.69 × 10^−11^	4.68 × 10^−2^	9.12 × 10^−1^	2.89 × 10^−3^	6.00 × 10^−1^	2.37 × 10^−10^	3.07 × 10^−11^
F21	3.02 × 10^−11^	6.20 × 10^−4^	8.10 × 10^−10^	6.52 × 10^−9^	7.77 × 10^−9^	3.02 × 10^−11^	3.02 × 10^−11^
F22	5.49 × 10^−11^	1.37 × 10^−1^	4.23 × 10^−3^	2.12 × 10^−1^	6.97 × 10^−3^	6.70 × 10^−11^	3.02 × 10^−11^
F23	3.02 × 10^−11^	3.34 × 10^−11^	3.02 × 10^−11^	2.07 × 10^−2^	6.74 × 10^−6^	3.02 × 10^−11^	3.02 × 10^−11^
F24	3.02 × 10^−11^	9.47 × 10^−1^	8.99 × 10^−11^	5.57 × 10^−10^	5.09 × 10^−6^	3.02 × 10^−11^	3.32 × 10^−11^
F25	3.02 × 10^−11^	3.02 × 10^−11^	3.02 × 10^−11^	3.02 × 10^−11^	3.02 × 10^−11^	3.02 × 10^−11^	3.02 × 10^−11^
F26	3.02 × 10^−11^	7.39 × 10^−11^	4.08 × 10^−11^	6.52 × 10^−1^	7.96 × 10^−1^	3.02 × 10^−11^	3.02 × 10^−11^
F27	3.02 × 10^−11^	7.39 × 10^−11^	3.02 × 10^−11^	6.72 × 10^−10^	1.11 × 10^−6^	3.02 × 10^−11^	3.02 × 10^−11^
F28	3.02 × 10^−11^	3.02 × 10^−11^	3.02 × 10^−11^	3.02 × 10^−11^	3.02 × 10^−11^	3.02 × 10^−11^	3.02 × 10^−11^
F29	3.02 × 10^−11^	3.02 × 10^−11^	3.02 × 10^−11^	4.08 × 10^−11^	4.23 × 10^−3^	3.02 × 10^−11^	3.42 × 10^−11^
F30	3.02 × 10^−11^	3.02 × 10^−11^	3.02 × 10^−11^	3.02 × 10^−11^	1.84 × 10^−2^	3.02 × 10^−11^	3.02 × 10^−11^

**Table 3:** Results of different swarm intelligence algorithms of CEC2020.

Function	Metric	SWDBO	DBO	PSO	BKA	GWO	HBA	WOA	BWO
F1	Best	7.95 × 10^7^	1.98 × 10^11^	3.77 × 10^10^	6.47 × 10^10^	2.85 × 10^10^	1.34 × 10^9^	4.16 × 10^10^	2.43 × 10^11^
F1	Std	1.32 × 10^8^	9.03 × 10^9^	1.41 × 10^10^	3.02 × 10^10^	1.21 × 10^10^	7.81 × 10^9^	9.22 × 10^9^	1.08 × 10^10^
F1	Avg	2.56 × 10^8^	2.24 × 10^11^	6.26 × 10^10^	1.10 × 10^11^	5.60 × 10^10^	1.50 × 10^10^	6.63 × 10^10^	2.83 × 10^11^
F2	Best	1.28 × 10^4^	2.88 × 10^4^	1.52 × 10^4^	1.54 × 10^4^	1.43 × 10^4^	1.63 × 10^4^	2.48 × 10^4^	3.13 × 10^4^
F2	Std	8.59 × 10^2^	1.38 × 10^3^	1.61 × 10^3^	3.01 × 10^3^	4.81 × 10^3^	4.41 × 10^3^	1.64 × 10^3^	4.84 × 10^2^
F2	Avg	1.48 × 10^4^	3.16 × 10^4^	1.83 × 10^4^	1.95 × 10^4^	1.85 × 10^4^	2.18 × 10^4^	2.80 × 10^4^	3.23 × 10^4^
F3	Best	2.22 × 10^3^	3.51 × 10^3^	2.67 × 10^3^	3.17 × 10^3^	1.88 × 10^3^	2.15 × 10^3^	3.32 × 10^3^	4.00 × 10^3^
F3	Std	2.70 × 10^2^	7.49 × 10^1^	3.17 × 10^2^	2.72 × 10^2^	1.60 × 10^2^	1.37 × 10^2^	1.73 × 10^2^	4.84 × 10^1^
F3	Avg	2.97 × 10^3^	3.69 × 10^3^	3.23 × 10^3^	3.42 × 10^3^	2.12 × 10^3^	2.48 × 10^3^	3.70 × 10^3^	4.10 × 10^3^
F4	Best	2.02 × 10^3^	1.20 × 10^7^	1.13 × 10^5^	7.77 × 10^4^	5.76 × 10^4^	3.94 × 10^3^	4.80 × 10^5^	1.09 × 10^8^
F4	Std	4.07 × 10^1^	4.60 × 10^6^	4.00 × 10^5^	1.59 × 10^7^	1.40 × 10^5^	5.12 × 10^3^	5.89 × 10^5^	5.50 × 10^7^
F4	Avg	2.08 × 10^3^	2.18 × 10^7^	6.41 × 10^5^	6.39 × 10^6^	2.26 × 10^5^	8.41 × 10^3^	1.18 × 10^6^	1.98 × 10^8^
F5	Best	5.20 × 10^6^	2.15 × 10^8^	1.30 × 10^7^	4.78 × 10^6^	2.51 × 10^7^	2.98 × 10^6^	5.07 × 10^7^	1.60 × 10^9^
F5	Std	2.66 × 10^6^	1.42 × 10^8^	2.98 × 10^7^	3.36 × 10^8^	2.26 × 10^7^	4.58 × 10^6^	4.97 × 10^7^	4.20 × 10^8^
F5	Avg	1.04 × 10^7^	4.80 × 10^8^	5.44 × 10^7^	1.31 × 10^8^	5.55 × 10^7^	1.04 × 10^7^	1.30 × 10^8^	2.49 × 10^9^
F6	Best	5.16 × 10^3^	1.21 × 10^4^	6.58 × 10^3^	7.20 × 10^3^	4.44 × 10^3^	4.48 × 10^3^	1.25 × 10^4^	3.29 × 10^4^
F6	Std	6.99 × 10^2^	1.75 × 10^3^	1.31 × 10^3^	4.25 × 10^3^	1.19 × 10^3^	7.23 × 10^2^	2.94 × 10^3^	3.43 × 10^3^
F6	Avg	6.50 × 10^3^	1.58 × 10^4^	8.47 × 10^3^	1.12 × 10^4^	5.73 × 10^3^	6.12 × 10^3^	1.77 × 10^4^	3.97 × 10^4^
F7	Best	2.09 × 10^6^	6.00 × 10^7^	3.66 × 10^6^	2.80 × 10^6^	4.71 × 10^6^	1.93 × 10^6^	3.71 × 10^7^	2.90 × 10^8^
F7	Std	1.00 × 10^6^	6.52 × 10^7^	9.29 × 10^6^	6.72 × 10^7^	1.32 × 10^7^	1.31 × 10^6^	3.87 × 10^7^	7.10 × 10^7^
F7	Avg	3.60 × 10^6^	2.03 × 10^8^	1.63 × 10^7^	2.45 × 10^7^	2.25 × 10^7^	4.33 × 10^6^	9.88 × 10^7^	4.83 × 10^8^
F8	Best	1.85 × 10^4^	2.71 × 10^4^	1.85 × 10^4^	1.82 × 10^4^	1.72 × 10^4^	1.76 × 10^4^	2.71 × 10^4^	3.26 × 10^4^
F8	Std	1.19 × 10^3^	1.92 × 10^3^	1.36 × 10^3^	3.11 × 10^3^	1.27 × 10^3^	3.60 × 10^3^	1.88 × 10^3^	5.56 × 10^2^
F8	Avg	2.02 × 10^4^	3.34 × 10^4^	2.13 × 10^4^	2.23 × 10^4^	1.95 × 10^4^	2.31 × 10^4^	3.01 × 10^4^	3.44 × 10^4^
F9	Best	4.06 × 10^3^	7.26 × 10^3^	5.19 × 10^3^	5.75 × 10^3^	4.15 × 10^3^	4.06 × 10^3^	5.81 × 10^3^	1.21 × 10^4^
F9	Std	6.06 × 10^2^	7.12 × 10^2^	4.86 × 10^2^	9.79 × 10^2^	1.25 × 10^2^	1.76 × 10^3^	4.33 × 10^2^	1.09 × 10^3^
F9	Avg	5.04 × 10^3^	8.28 × 10^3^	6.10 × 10^3^	6.90 × 10^3^	4.38 × 10^3^	5.37 × 10^3^	6.52 × 10^3^	1.45 × 10^4^
F10	Best	3.40 × 10^3^	1.69 × 10^4^	5.58 × 10^3^	7.07 × 10^3^	5.31 × 10^3^	3.84 × 10^3^	6.40 × 10^3^	3.09 × 10^4^
F10	Std	7.07 × 10^1^	1.28 × 10^3^	9.77 × 10^2^	5.61 × 10^3^	8.03 × 10^2^	4.67 × 10^2^	7.94 × 10^2^	1.00 × 10^3^
F10	Avg	3.61 × 10^3^	1.93 × 10^4^	7.85 × 10^3^	1.24 × 10^4^	6.43 × 10^3^	4.39 × 10^3^	7.76 × 10^3^	3.29 × 10^4^

**Table 4:** Comparison of Wilcoxon rank-sum results for CEC2020.

Function	DBO	PSO	BKA	GWO	HBA	WOA	BWO
F1	3.02 × 10^−11^	3.02 × 10^−11^	3.02 × 10^−11^	3.02 × 10^−11^	3.02 × 10^−11^	3.02 × 10^−11^	3.02 × 10^−11^
F2	3.43 × 10^−11^	1.46 × 10^−10^	6.07 × 10^−11^	3.50 × 10^−9^	3.34 × 10^−11^	3.02 × 10^−11^	3.03 × 10^−11^
F3	3.02 × 10^−11^	2.27 × 10^−3^	1.01 × 10^−8^	6.07 × 10^−11^	9.26 × 10^−9^	3.34 × 10^−11^	3.32 × 10^−11^
F4	3.02 × 10^−11^	3.02 × 10^−11^	3.02 × 10^−11^	3.02 × 10^−11^	3.02 × 10^−11^	3.02 × 10^−11^	3.02 × 10^−11^
F5	3.23 × 10^−11^	4.98 × 10^−11^	4.74 × 10^−6^	3.02 × 10^−11^	6.31 × 10^−1^	3.02 × 10^−11^	3.02 × 10^−11^
F6	3.02 × 10^−11^	1.56 × 10^−8^	4.50 × 10^−11^	3.37 × 10^−5^	5.94 × 10^−2^	3.02 × 10^−11^	3.36 × 10^−11^
F7	3.02 × 10^−11^	2.87 × 10^−10^	6.53 × 10^−7^	4.50 × 10^−11^	2.92 × 10^−2^	3.02 × 10^−11^	3.01 × 10^−11^
F8	3.08 × 10^−11^	1.95 × 10^−3^	2.53 × 10^−4^	1.58 × 10^−1^	3.59 × 10^−5^	3.02 × 10^−11^	3.32 × 10^−11^
F9	3.02 × 10^−11^	6.53 × 10^−8^	2.15 × 10^−10^	3.57 × 10^−6^	1.45 × 10^−1^	1.78 × 10^−10^	3.02 × 10^−11^
F10	3.02 × 10^−11^	3.02 × 10^−11^	3.02 × 10^−11^	3.02 × 10^−11^	3.02 × 10^−11^	3.02 × 10^−11^	3.42 × 10^−11^

**Table 5:** Comparison table of optimization results for three-bar truss design.

Three-Bar Truss Design	SWDBO	DBO	PSO	BKA	GWO	HBA	WOA	BWO
Best	2.64 × 10^2^	2.64 × 10^2^	2.64 × 10^2^	2.64 × 10^2^	2.64 × 10^2^	2.64 × 10^2^	2.64 × 10^2^	2.64 × 10^2^
Worst	2.64 × 10^2^	2.64 × 10^2^	2.64 × 10^2^	2.64 × 10^2^	2.64 × 10^2^	2.64 × 10^2^	2.66 × 10^2^	2.67 × 10^2^
Std	1.43 × 10^−3^	1.41 × 10^−1^	8.89 × 10^−3^	5.99 × 10^−3^	8.79 × 10^−3^	1.24 × 10^−4^	7.59 × 10^−1^	8.26 × 10^−1^
Mean	2.64 × 10^2^	2.64 × 10^2^	2.64 × 10^2^	2.64 × 10^2^	2.64 × 10^2^	2.64 × 10^2^	2.65 × 10^2^	2.65 × 10^2^
Median	2.64 × 10^2^	2.64 × 10^2^	2.64 × 10^2^	2.64 × 10^2^	2.64 × 10^2^	2.64 × 10^2^	2.64 × 10^2^	2.65 × 10^2^
Time	3.38 × 10^−1^	1.29 × 10^−1^	8.85 × 10^−2^	3.15 × 10^−1^	1.03 × 10^−1^	1.21 × 10^−1^	1.01 × 10^−1^	9.15 × 10^−1^

**Table 6:** Comparison table of optimization results for ten-bar truss design.

Ten-Bar Truss Design	SWDBO	DBO	PSO	BKA	GWO	HBA	WOA	BWO
Best	5.25 × 10^2^	5.36 × 10^2^	5.26 × 10^2^	5.24 × 10^2^	5.25 × 10^2^	5.25 × 10^2^	6.14 × 10^2^	6.36 × 10^2^
Worst	5.32 × 10^2^	5.87 × 10^2^	5.80 × 10^2^	5.31 × 10^2^	5.32 × 10^2^	5.35 × 10^2^	7.66 × 10^2^	7.68 × 10^2^
Std	2.40 × 10^0^	1.78 × 10^1^	1.62 × 10^1^	1.93 × 10^0^	2.63 × 10^0^	3.08 × 10^0^	5.46 × 10^1^	4.36 × 10^1^
Mean	5.27 × 10^2^	5.59 × 10^2^	5.40 × 10^2^	5.25 × 10^2^	5.28 × 10^2^	5.28 × 10^2^	6.83 × 10^2^	7.04 × 10^2^
Median	5.27 × 10^2^	5.53 × 10^2^	5.33 × 10^2^	5.24 × 10^2^	5.28 × 10^2^	5.27 × 10^2^	6.57 × 10^2^	7.05 × 10^2^
Time	2.64 × 10^0^	1.48 × 10^0^	1.41 × 10^0^	3.01 × 10^0^	1.39 × 10^0^	1.56 × 10^0^	1.51 × 10^0^	3.84 × 10^0^

**Table 7:** Comparison table of optimization results for welding beam design.

Welded Beam	SWDBO	DBO	PSO	BKA	GWO	HBA	WOA	BWO
Best	1.34 × 10^0^	1.34 × 10^0^	1.34 × 10^0^	1.34 × 10^0^	1.34 × 10^0^	1.34 × 10^0^	1.38 × 10^0^	1.38 × 10^0^
Worst	1.34 × 10^0^	1.34 × 10^0^	1.34 × 10^0^	1.34 × 10^0^	1.34 × 10^0^	1.34 × 10^0^	1.70 × 10^0^	1.50 × 10^0^
Std	6.89 × 10^−6^	2.46 × 10^−4^	9.72 × 10^−5^	2.24 × 10^−5^	7.29 × 10^−5^	1.32 × 10^−5^	1.04 × 10^−1^	4.13 × 10^−2^
Mean	1.34 × 10^0^	1.34 × 10^0^	1.34 × 10^0^	1.34 × 10^0^	1.34 × 10^0^	1.34 × 10^0^	1.57 × 10^0^	1.46 × 10^0^
Median	1.34 × 10^0^	1.34 × 10^0^	1.34 × 10^0^	1.34 × 10^0^	1.34 × 10^0^	1.34 × 10^0^	1.59 × 10^0^	1.47 × 10^0^
Time	1.98 × 10^−1^	6.29 × 10^−2^	3.14 × 10^−2^	1.68 × 10^−1^	3.96 × 10^−2^	5.76 × 10^−2^	3.29 × 10^−2^	6.48 × 10^−1^

## Data Availability

The data that support the findings of this study are available from the corresponding author upon request. There are no restrictions on data availability.
